# Local oxygen tension dictates hematopoietic cell growth and potency

**DOI:** 10.1038/s41375-026-02971-w

**Published:** 2026-05-15

**Authors:** James Ropa, Sarah Gutch, Lindsay Wathen, So Jeong Kim, Jimin Park, Jessica Newton, Gracie Whitacre, Arafat Aljoufi, Scott Cooper, Wouter Van’t Hof, Mark H. Kaplan, Maegan L. Capitano

**Affiliations:** 1 Department of Medical and Molecular Genetics, Indiana University School of Medicine, Indianapolis, IN USA; 2 Department of Microbiology and Immunology, Indiana University School of Medicine, Indianapolis, IN USA; 3 Cleveland Cord Blood Center, Cleveland, OH USA

**Keywords:** Haematopoietic stem cells, Haematopoiesis, Bone marrow transplantation

## Abstract

Hematopoietic stem and progenitor cells support a lifetime supply of blood and immune cells and constitute a powerful cell therapy platform for hematologic diseases. In this study, we define oxygen-dependent activities of hematopoietic stem and progenitor cells. We found that lineage-defined progenitor cells from umbilical cord blood, bone marrow, and mobilized peripheral blood showed increased expansion in high oxygen, while primitive cells- including those from cord blood with in vivo potency- were maintained at higher frequencies in low physiologic O_2_. Single cell transcriptomic profiling of hematopoiesis under varying oxygen revealed the expected modulation of molecular hypoxia programs, including HIF and MTORc signaling. Transcriptomics also identified genes that are understudied in the context of oxygen dependency including MDM4 pathway and *PRSS2*, which we found is an mRNA biomarker for hematopoietic cell potency. Transcriptional changes together with biochemical validation revealed that low oxygen preserves cells with lower metabolic activity in a less proliferative state that exhibit decreased accumulation of stress markers. This likely occurs via dynamic interplay of multiple molecular programs and may drive differences in cell potency. Collectively, these data identify oxygen-sensing pathways as targets to improve cell therapies and suggest that local oxygenation dictates hematopoietic potential in anatomic niches.

## Introduction

Hematopoietic stem (HSCs) and progenitor cells (HPCs) are responsible for consistent replenishment of blood and immune cells throughout human life, are a source of life-saving treatment in the form of cell therapies, and are dysregulated in various diseases including malignancy [1, 2]. A comprehensive understanding of intrinsic and extrinsic factors that affect the function and potency of HSCs/HPCs is still desired [3, 4]. Oxygen (O_2_) is an extrinsic environmental factor with the potential to affect cellular function [5]. Different hematopoietic niches exist at variable local O_2_ tensions, which are also hypoxic compared to ambient air [6–11]. Many tissues, such as bone marrow (BM) exhibit gradients of oxygenation within the same niche [6–11]. Understanding how the various O_2_ tensions that HSCs/HPCs are exposed to throughout their lifespan affects their function will provide insights into their regulation and represents a potential tool to modify their function. Acute exposure to very low O_2_ availability (hypoxia) is well known to affect the health of cells [12]. Regional hypoxia has become an area of great interest in the field of tumor biology, as variable O_2_ levels affect tumor growth, metastasis, and responses to therapies [13, 14]. Further, work by our group has shown that exposure to extra physiologic O_2_ that occurs when cells are manipulated ex vivo negatively impacts HSC function [15–19]. If and how the entire range of physiologic and non-physiologic O_2_ tensions that hematopoietic cells are exposed to affects their growth and function requires further elucidation.

Factors affecting cell potency also have important implications for hematopoietic cell therapies, which utilize hematopoietic cells isolated from BM, mobilized peripheral blood (mPB), or umbilical cord blood (CB) to treat patients with hematologic disorders [20]. For CB in particular, cell potency (the ability of the cells to repopulate a healthy hematopoietic system), is a critical limiting factor for efficacious treatment [4, 21–23]. Improving the potency of HSCs/HPCs with treatments or ex vivo expansion may enhance the efficacy of CB transplantation and other hematopoietic cell therapies, such as gene editing, that require ex vivo manipulation [24, 25]. Expansion of HSCs/HPCs under hypoxia has been studied as a possible way to improve cell potency, but identifying the optimal O_2_ tension and elucidating the heterogeneous effect that varying O_2_ levels has on HSCs/HPCs has remained challenging [26–29].

In this study, we hypothesized that cells utilize variable local O_2_ tensions to perform distinct functions and examined how a wide range of oxygenation affects the phenotypic, functional, expansion, and molecular characteristics of primary human CD34+ cells, which are enriched for HSCs/HPCs. Our findings can be used to improve hematopoietic cell therapies and provide insight into O_2_ as a critical regulator of hematopoietic cell function in different anatomic niches.

## Methods

### Ethics statement

All methods were performed in accordance with Indiana University guidelines and regulations. All animal studies were approved by the Indiana University IACUC. Human umbilical cord blood, bone marrow, and peripheral blood samples were purchased as research units, were fully de-identified, and are not considered human subjects research.

### Umbilical cord blood primary samples

Cord blood units (CBU) were collected and distributed under the supervision of Cleveland Cord Blood Center. CD34+ cells were enriched within 55 h of birth using density gradient centrifugation and Miltenyi enrichment kits.

### Ex vivo expansion in variable oxygen

5×10^4^ CD34+ cells were plated in SFEM II serum free media supplemented with 100 ng/mL human TPO, FLT3L, and SCF. Cultures were incubated at 37°C, 5% CO_2_ and 1%, 3%, 5%, 14%, or 21% O_2_.

### Mouse model of transplantation

All mouse work was approved by the Indiana University IACUC. 24 h prior to transplantation, 10 week old female NOD-scid IL2Rgamma^null^ (NSG) were sublethally irradiated (350gy). Mice were transplanted by tail vein injection with 2.5×10^4^ freshly thawed unmanipulated CD34+ enriched cells or 2.5×10^4^ CD34+ cells plus all expanded progeny. 9 mice split across 3 CBUs were used for each condition. Five mice died in the course of the experiment due to technical husbandry issues that were unrelated to experimental conditions and were unable to be included in later time point analyses. All samples were matched across conditions, therefore no mouse randomization was necessary or used. No blinding was performed.

### RNA-sequencing

Bulk RNA was harvested using the Qiagen RNeasy Micro Plus Kit. Libraries were prepared using the SMART-Seq v4 Ultra Low Input RNA Kit (Takara) or the NEBNext Ultra Low Input RNA Sequencing Kit (NEB). Single cells expanded in low O_2_ (1%, 3%, and 5%) were harvested in a hypoxia chamber equilibrated to 3% O_2_. Unmanipulated cells and those expanded at high O_2_ tensions (14% and 21%) were harvested ambient air. Cells were hashed by condition. Using the PIPseq T20 kit (Fluent Biosciences), 5×10^4^ cells from each CBU was subjected to single cell barcoding using particle emulsions and libraries were prepared according to manufacturer’s recommendations [30]. Sequencing was performed by the IU Center for Medical Genomics.

### Statistics

Sample sizes for each experiment were chosen based on power analyses, availability of donor samples/cells, and previous experience of the lead author. Variance was similar across groups.

## Results

### Hematopoietic cells from differently oxygenated niches exhibit distinct properties

**Fig. 1:**
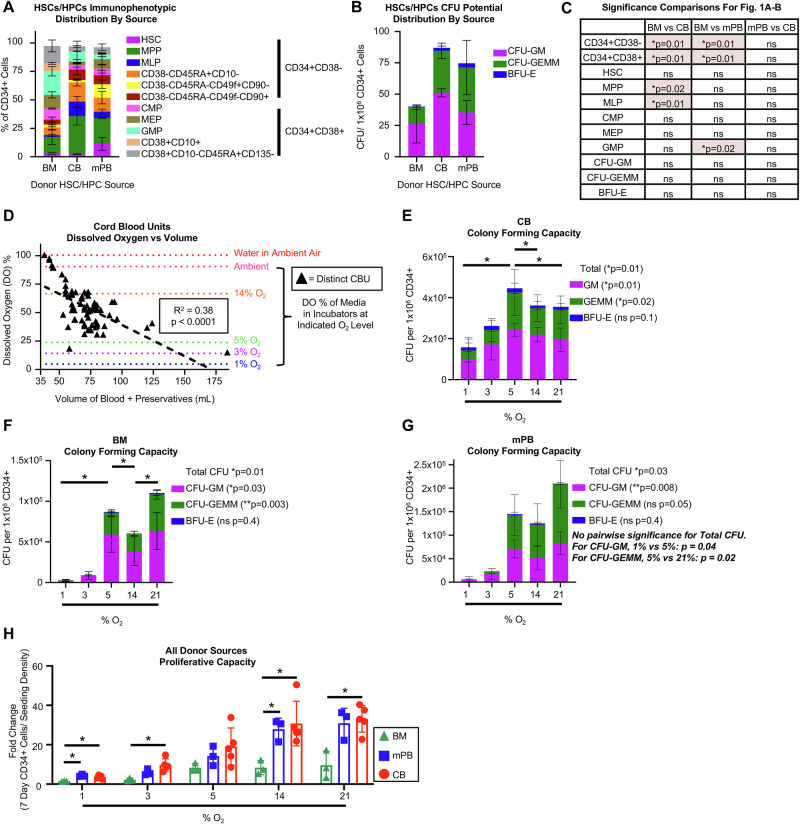
HSCs/HPCs display O_2_ dependencies. **A** Immunophenotypically defined HSCs/HPCs or **B** CFU numbers as a fraction of CD34+ cells from BM (*n* = 3), mPB (*n* = 3), and CB (*n* = 2). **C** Significance analysis of A using 1-way ANOVA with post hoc comparisons. **D** Dissolved O_2_ concentration in fresh whole CBUs compared to total volume of the CBU. Dissolved O_2_ levels associated with physiologically relevant local and atmospheric O_2_ tensions are indicated by dashed lines. **E–G** CFU from CB (*n* = 8), BM (*n* = 3), or mPB (*n* = 3) CD34+ cells performed in the indicated O_2_ tensions. 12 days after plating CFUs of different potentials were enumerated manually by counting. **H** CB (*n* = 5), BM (*n* = 3), or mPB (*n* = 3) CD34+ cells expanded in serum free media with growth factors for 7 days in the indicated O_2_ tensions. Total CD34+ cell numbers compared to the number of cells seeded by flow cytometry analysis. Stats: one-way ANOVA controlling for donor unit (indicated by p-value) with post hoc Tukey or Sidak comparisons. *p < 0.05. HSCs hematopoietic stem cells; HPCs hematopoietic progenitor cells; CFU colony forming unit; BM bone marrow; mPB mobilized peripheral blood; CB umbilical cord blood; O_2_ oxygen.

Each hematopoietic niche exists at different O_2_ tensions (Table 1) [6–11]. The BM ranges from <1–6% O_2_ while peripheral blood (PB) total oxygenation lies between 4 and 14% (with location dependent levels of hemoglobin-bound or dissolved O_2_ [31]) and is different in arterial or venous blood. CB exhibits ranges from 0.5–6% O_2_ [7, 8], though possibly up to 10% (this study, Fig. 1D). Other tissues where HSCs/HPCs reside like the lung are highly oxygenated, up to 17% [11, 32]. We infer that HSC/HPC responses to O_2_ could be a consequence of specialization of the tissue they reside in. To test differences in HSCs/HPCs from differently oxygenated niches, we performed ex vivo characterization of HSCs/HPCs from human CB, mPB, or BM CD34+ cells using immunophenotypic analysis by cell surface staining with flow cytometry and colony forming unit (CFU) assays (Fig. 1A–C) [33, 34]. This showed that the HSC/primitive HPC enriched CD34 + CD38- fraction is higher in mPB and CB compared to BM (Fig. 1A). There was also a significantly higher frequency of multipotent progenitors (MPPs) and multilymphoid progenitors (MLPs) in CB compared to BM (Fig. 1A, B). Conversely, BM contains a higher proportion of more lineage determined CD34 + CD38+ HPCs, with a significantly higher granulocyte-macrophage progenitors (GMPs) frequency compared to mPB (Fig. 1A, B). CB and mPB show increased trends of functional HPCs, demonstrated by numbers of CFU-granulocyte, macrophage (CFU-GM) and CFU-granulocyte, erythroid, macrophage, megakaryocyte (CFU-GEMM) compared to BM (Fig. 1B, C). These data suggest that CD34+ cells exhibit different proportions of HSCs/HPC subpopulations when isolated from different niches.

**Table 1:** O_2_ Tensions of Hematopoietic Tissues.

Tissue	Reported O_2_	Citations	Hematopoietic niche?
**Bone marrow**	1-6% (animal models)	Spencer, et al. 2014 [4]	Yes
**Peripheral blood**	6.7%-13%	Honarmand & Safavi 2008 [5]	Yes
	4.3%-14%	Byrne, et a.l 2014 [6]	
**Umbilical cord blood**	0-10% (mean 3%)	This Study (Fig. 1D)	Yes
	0.6-6%	Saneh, et al. 2023 [7], Arikan, et al. 2000 [8]	
**Lung**	10.5%-16.4%	Hamedani, et al. 2016 [9]	Yes

Shown are modeled or measured O_2_ tensions in various hematopoietic niches as reported in the literature. The values from “This Study” were calculated based on the dissolved O_2_ content of umbilical cord blood, accounting for dilution of the cord blood hypoxic state that occurs when preservatives are added to the total blood volume (Fig. 1D).

We have previously suggested that donor hematopoietic cells used in therapy and research are necessarily exposed to ambient O_2_ levels upon harvest and thus lose their relative hypoxic states [15–17, 35]. To test this directly, we measured the dissolved O_2_ concentration in > 70 fresh CBUs that were never frozen and were measured within 1 week of isolation. To our surprise, the dissolved O_2_ level of CBUs collected in preservation bags remains hypoxic relative to fully oxygenated liquids (Fig. 1D). The level of dissolved O_2_ directly correlates with the volume of the unit. This is likely the result of the physiologic oxygenation state of the blood being slightly diluted by the addition of fully oxygenated anti-coagulant preservatives in each CBU, which impacts low collection volumes the most. Thus, CB remains lowly oxygenated after collection. We thus focus on CB as a primary model for studying O_2_ dependency. We also examine BM and mPB to explore differential O_2_ sensitivity of HSCs/HPCs from different donor sources, inferring that their exposure to extra physiologic O_2_ is likely similar to that of CB.

### Hematopoietic cells exhibit O_2_ dependency ex vivo

Differently oxygenated hematopoietic niches have non-O_2_ related environmental factors that could account for changes to cellular properties. We thus sought to test the effects of O_2_ on hematopoietic cell growth and function in a single variable system using oxygen-controlled incubators. We maintained CD34+ cells in 1%, 3%, 5%, 14%, and 21% O_2_, which spans the range of O_2_ levels in tissues in which hematopoietic cells reside, migrate through, or are processed (Table 1). First, we measured the CFU potential of the CD34+ cell fraction and found that total CFU, CFU-GM, and CFU-GEMM numbers from CB, BM, and mPB are significantly altered by local O_2_ tension. Total CB CFU numbers are significantly higher in 5% O_2_ compared to 14% and 21% and CB CFU-GM numbers are higher in 5% compared to 1% (Fig. 1E), while BM and mPB CFU numbers show direct correlation to O_2_ tension, with increased CFU-GM, CFU-GEMM, and total CFU at 5%, 14%, and 21% O_2_ compared to lower tensions (Fig. 1F, G). Burst forming unit-erythroid (BFU-E) numbers were unaffected by variable O_2_. Next, we cultured CD34+ cells in serum free media with growth factors for 7 days to measure their proliferative capacity. CB and mPB CD34+ cells exhibit increased proliferation with increased O_2_ tension (Fig. 1H). Interestingly, at all tensions except 5% O_2_, CD34+ cells from mPB and/or CB exhibited significantly higher proliferation compared to BM.

### Multipotent hematopoietic cells exhibit preferential expansion in different O_2_ ranges compared to lineage determined HPCs

**Fig. 2:**
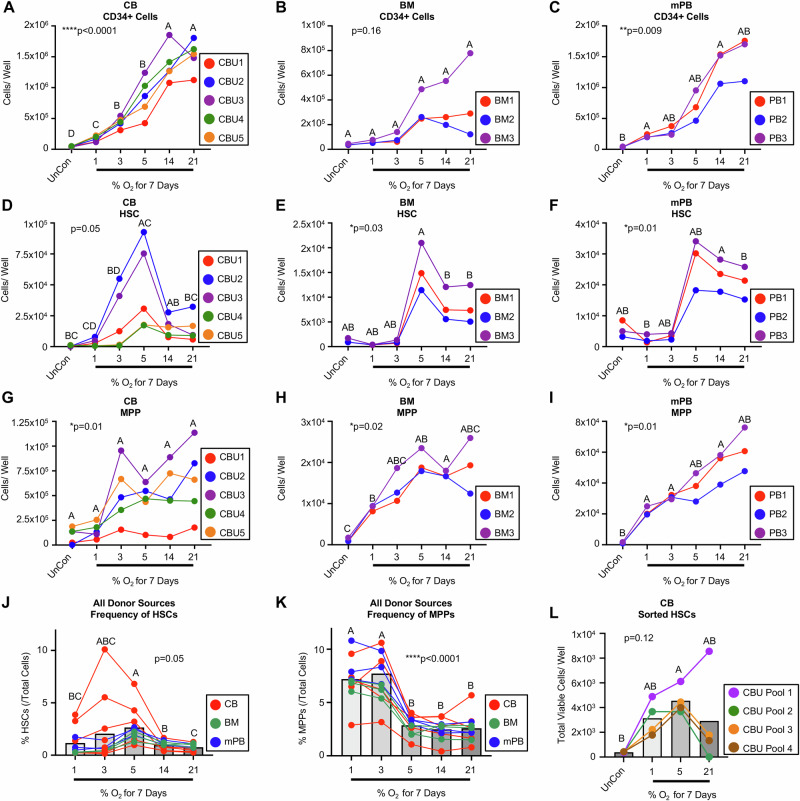
Multipotent HSCs/MPPs expand the most in low physiologic O_2_ tension. **A–I** CB (*n* = 5), BM (*n* = 3), or mPB (*n* = 3) CD34+ cells were expanded in serum free media with growth factors for 7 days in the indicated O_2_ tensions. Cells were then analyzed for enumeration of total numbers of the indicated immunophenotypically defined population by flow cytometry. **J**, **K** Percent of indicated immunophenotypic HSC/HPC population as a fraction of total cells in the well after 7 days of expansion. **L** CB HSCs were sorted prospectively from CD34+ cells prior to any culture. Due to cell number limitations, HSC sorted populations were pooled so that each expansion was seeded as a pool of two distinct CB units. Sorted pools were then expanded in serum free media with growth factors for 7 days in the indicated O_2_ tensions. Cells were analyzed for total viable cell expansion by counting using a hemacytometer (*n* = 4 CB pools). Two independent replicates from the HSC analysis were excluded due to detection of a contaminating cell population. This contamination was identified by outlier levels of total expansion (over 200-fold expansion specifically in 5%) combined with flow cytometry confirming 0% CD34+ cells in the expanded cell population. Stats: one-way ANOVA (indicated by p-value) with post hoc comparisons indicated by compact letter display (in compact letter display, groups that are not statistically different are indicated by matching letters). Each point/color indicates distinct biological donor sources as indicated by the included legends. HSCs hematopoietic stem cells; HPCs hematopoietic progenitor cells; CFU colony forming unit; BM bone marrow; mPB mobilized peripheral blood; CB umbilical cord blood; O_2_ oxygen; UnCon Unmanipulated control (input).

CD34+ cells are heterogenous, so we next used immunophenotypic analysis following culture to determine how different HSC/HPC subpopulations expand in variable O_2_. For cell fractions derived from CB and mPB but not BM, total nucleated cells, total CD34+ cells and the multipotent cell enriched CD34 + CD38- fraction, and the lineage defined progenitor enriched CD34 + CD38+ fraction exhibited significantly increased expansion in increased O_2_ with higher total cell numbers at 5%, 14%, and 21% O_2_ compared to 1%, 3% and input (Fig. 2A–C; Fig. S1A–I). HSC growth was significantly affected by O_2_ tension with peak expanded cell numbers in 5% O_2_ in cells from mPB and BM, while HSCs from CB exhibited a similar trend (Fig. 2D–F). MPPs from all sources are also sensitive to changing O_2_, but the ideal growth oxygenation varies between CB, BM, and mPB (Fig. 2G–I). Interestingly, when combining all donor sources HSCs exhibit the highest frequency of HSCs in 5% O_2_, while MPP frequencies are highest in 1–3%, suggesting these cell types preferentially proliferate in different local O_2_ levels (Fig. 2J, K). An important caveat to immunophenotyping expanded cell populations is that cell surface markers may change in culture or as a result of changing local oxygenation. We thus sorted immunophenotypically defined HSCs/HPCs from CB prior to any ex vivo cell culture and seeded these pure populations in serum free media with growth factors at 1%, 5%, or 21% O_2_. Consistent with the previous results, only 5% O_2_ supported significant expansion compared to unmanipulated control input cells (Fig. 2L), and sorted MPPs showed trends toward higher expansion with higher oxygenation (Fig. S2A).

**Fig. 3:**
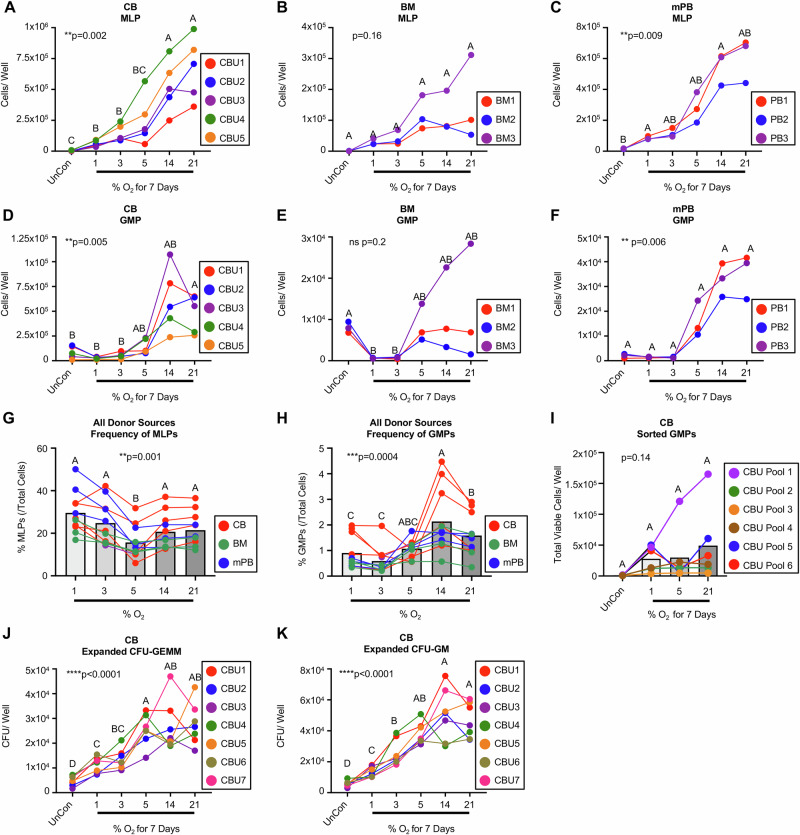
Lineage committed HPCs exhibit differential preferences for O_2_ tension. **A–F** CB (*n* = 5), BM (*n* = 3), or mPB (*n* = 3) CD34+ cells were expanded in serum free media with growth factors for 7 days in the indicated O_2_ tensions. Cells were then analyzed for enumeration of total numbers of the indicated immunophenotypically defined population by flow cytometry. **G**,**H** Percent of indicated immunophenotypic HPC population as a fraction of total cells in the well after 7 days of expansion. **I** Cord blood (CB) GMPs were sorted prospectively from CD34+ cells prior to any culture. Due to cell number limitations, GMP sorted populations were pooled so that each expansion was seeded as a pool of two distinct CB units. Sorted pools were then expanded in serum free media with growth factors for 7 days in the indicated O_2_ tensions. Cells were analyzed for total viable cell expansion by counting using a hemacytometer (*n* = 6 CB pools). **J, K** CB CD34+ cells (*n* = 7) were expanded in serum free media with growth factors for 7 days in the indicated O_2_ tensions. Cells were then plated in CFU assays at the same tension (5%). 12 days after plating CFUs of different potentials were enumerated manually by counting. Stats: one-way ANOVA (indicated by p-value) with post hoc comparisons indicated by compact letter display (in compact letter display, groups that are not statistically different are indicated by matching letters). Each point/color indicates distinct biological donor sources as indicated by the included legends. HSCs hematopoietic stem cells; HPCs hematopoietic progenitor cells; CFU colony forming unit; BM bone marrow; mPB mobilized peripheral blood; CB umbilical cord blood; O_2_ oxygen; UnCon Unmanipulated control (input).

Lineage defined progenitors including MLPs, GMPs, and common myeloid progenitors (CMPs) expanded significantly more in high O_2_ tensions with CB showing the significantly higher expansion in 5-21% compared to lower tensions with similar trends in BM and mPB (Fig. 3A–F, Fig. S1J–L). Megakaryocyte-erythroid progenitor (MEP) expansion was not significantly affected by O_2_ tension (Fig. S1M–O). In combining all donor sources, frequencies of MLPs were lowest in 5% O_2_ but similar in all other tensions, while GMP proportions were highest in 14-21% O_2_, showing still different O_2_ dependencies from HSCs/MPPs (Fig. 3G, H). Sorted lineage defined MEPs showed significantly higher expansion in 21% O_2_ compared to lower tensions, and other HPCs including GMPs, MLPs, and CMPs exhibit a similar trend consistent with total CD34+ expansions (Fig. 3I, S2B–D). Interestingly, 1% O_2_ almost universally allowed seeded cells to retain the highest percent of initial immunophenotype (Fig. S2E–J). For example, sorted MPPs yielded the highest frequency of MPPs after 7 days of expansion in 1%. HSCs were the sole exception, which were best maintained even proportionally in 5% O_2_. To test the function of expanded HPCs, we performed CFU assays on CD34+ cells expanded from CB. CFU-GM and CFU-GEMM both expanded more in liquid culture at 5, 14, and 21% compared to lower O_2_ tensions (Fig. 3J, K). Together, these data show that immunophenotypic mature progenitors and functional CFUs exhibit increased expansion in higher O_2_ levels, though responses vary slightly with donor source. Importantly, HSCs and more primitive HPCs maintain higher proportional representation in the expanded pool of cells in low physiologic O_2_, though this varies by cell type.

### CD34+ expansion in variable O_2_ affects in vivo hematopoietic engraftment

**Fig. 4:**
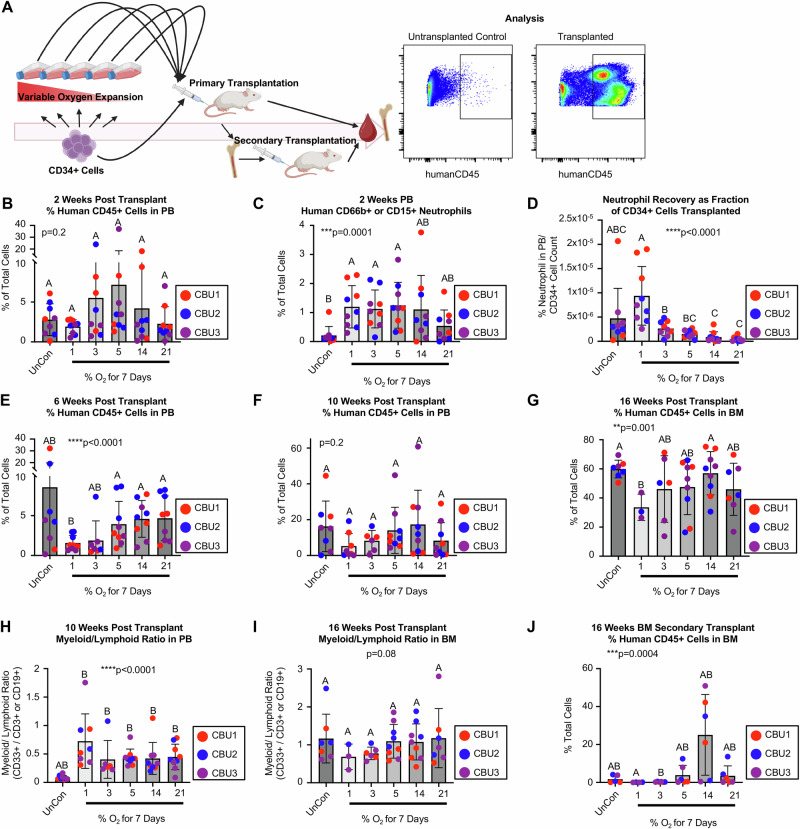
Expanded HSC/HPC in vivo potency is affected by local O_2_ tension. **A** Experimental scheme for in vivo studies. **B–J** CB CD34+ (*n* = 3) cells expanded in serum free media with growth factors for 7 days in the indicated O_2_ tensions. Input cells (25,000/ mouse) or input cells plus expanded progeny were transplanted to sublethally irradiated NSG mice (*n* = 9 each group). **B**,**C** Mice were monitored at 2 weeks for total human CD45+ chimerism and neutrophil recovery in PB by flow cytometry. **D** Neutrophil recovery percentage as a fraction of total cells transplanted (25,000 input cells + expanded progeny). **E**,**F** Mice were monitored at 6 and 10 weeks for total human CD45+ chimerism in PB by flow cytometry. **G** Mice were monitored at 16 weeks for total human CD45+ chimerism in BM by flow cytometry. **H**,**I** Mice were monitored at 10 weeks in the PB and 16 weeks in the BM for myeloid/lymphoid recovery by flow cytometry. Myeloid cells were defined by CD33+ cells while lymphoid cells were positive for CD3 and/or CD19. **J** Pooled BM cells from the primary transplant recipients were injected into sublethally irradiated secondary recipients (*n* = 5 each group). Engraftment was monitored at 16 weeks post transplantation in the BM of secondary recipients. Stats: one-way ANOVA (indicated by p-value) with post hoc comparisons indicated by compact letter display (in compact letter display, groups that are not statistically different are indicated by matching letters). Each color indicates distinct biological donor sources as indicated by the included legends. Each point indicates a recipient mouse. Notes on mouse numbers: the following groups lost mice due to experimental conditions (likely bone marrow failure or infection) during the course of the transplantation experiment: 1%- 1 mouse; 3%- 3 mice; 5%- 0 mice; 14%- 0 mice; 21%- 2 mice. 5 mice were lost from the 1% group due to husbandry complications unrelated to the experimental conditions. HSCs hematopoietic stem cells; HPCs hematopoietic progenitor cells; CFU colony forming unit; BM bone marrow; PB peripheral blood; CB umbilical cord blood; O_2_ oxygen; UnCon Unmanipulated control (input).

Ex vivo analyses do not always recapitulate the in vivo function of human HSCs/HPCs, thus in vivo mouse models are required for full evaluation of potency. We examined the ability of cells expanded at variable O_2_ levels to repopulate a hematopoietic system in vivo (Fig. 4A). CD34+ cells were expanded in five O_2_ tensions for 7 days in serum free media with growth factors. Unmanipulated “input” cells (2.5×10^4^ cells) or input cells plus their expanded progeny (variable numbers based on total cell expansion) were transplanted to NSG mice. While total human engraftment was not significantly different at 2 weeks post transplantation (Fig. 4B), early human neutrophil recovery measured in mouse PB, an analogous measure of successful patient transplantation, was increased when cells were expanded in low physiologic O_2_ tensions (1-5%) compared to input cells (Fig. 4C). When correcting for differential cell numbers transplanted due to differential expansion, early neutrophil recovery as a fraction of transplanted CD34+ cells was significantly higher in 1% O_2_ than in all higher tensions (Fig. 4D). 6 weeks post transplantation, mice transplanted with cells expanded at 1% O_2_ showed lower total engraftment in the PB compared to higher tensions (Fig. 4E), a transient effect no longer observed by 10 weeks post-transplantation (Fig. 4F), but which remained a trend in the BM at 16 weeks post transplantation (Fig. 4G). At 10 weeks, mice transplanted with expanded cells at all O_2_ tensions had significantly higher human myeloid/lymphoid ratio in their PB compared to those transplanted with unmanipulated input cells, with mice transplanted with cells expanded at 1% trending toward the most “balanced” ratio (Fig. 4H). No differences in myeloid/lymphoid balance were observed in the BM 16 weeks post transplantation, indicating either a transient effect or one that can only be observed in the PB (Fig. 4I). In secondary engraftment assays, CD34+ cells initially expanded at 14% O_2_ yielded the highest engraftment and was the only condition where all mice had observable secondary engraftment (Fig. 4J, data not shown). Together, these data show that different cell populations are responsible for different engraftment kinetics, such as early myeloid recovery and long-term reconstitution, and that these subpopulations are variably affected by physiologic levels of O_2_.

### Expansion in variable O_2_ alters the hematopoietic transcriptome

**Fig. 5:**
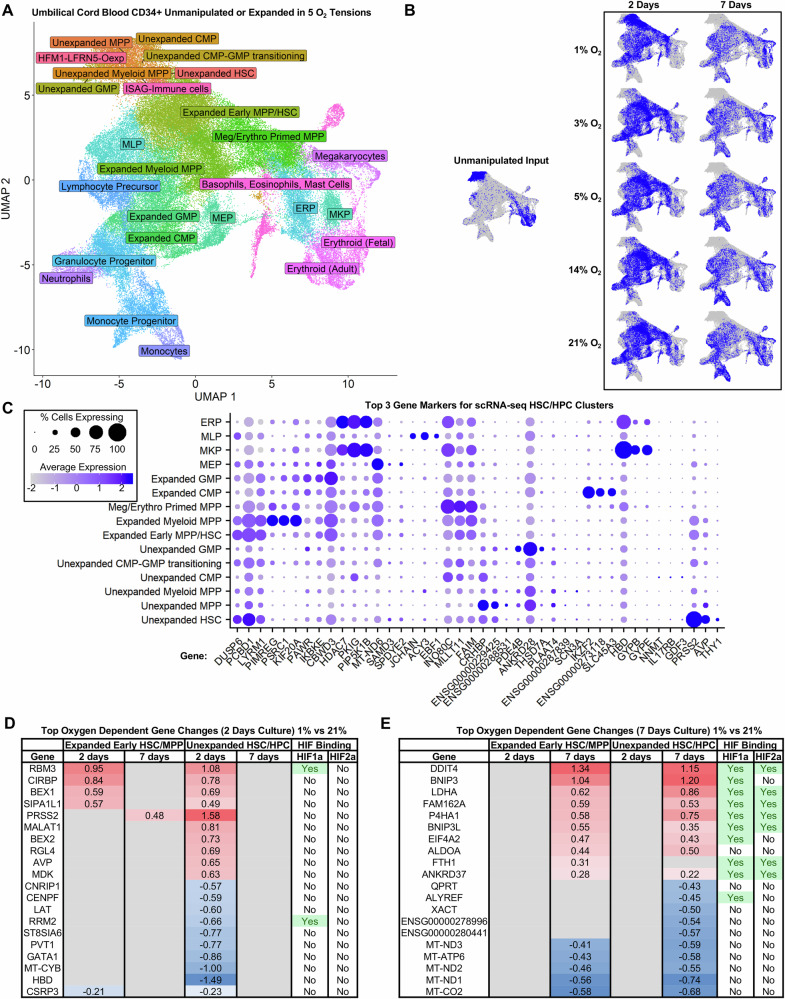
scRNA-seq of HSCs/HPCs reveals markers of potent HSCs/MPPs. **A–E** CB CD34+ cells expanded in serum free media with growth factors for 2 days (*n* = 2) or 7 days (*n* = 2) in the indicated O_2_ tensions, or unmanipulated input cells (*n* = 2) were subjected to scRNA-seq using particle-templated instant partition sequencing (PIP-seq). **A** UMAP showing annotated clusters from all cells and conditions. **B** UMAP plots with cells from the indicated expansion condition highlighted. **C** Dot Plot showing top three gene markers used to annotate HSC/HPC clusters. **D**,**E** Top differentially expressed genes from the comparison of 1 vs 21% for the indicated cell populations after 2 days (**D**) or 7 days (E) in culture. Annotated are the genes with known proximal HIF binding sites in hematopoietic cell lines. Genes are only shown if they were significantly different (p_adj_<0.05) in pseudobulked differential expression analysis. The full list of genes can be found in Table S2. HSCs hematopoietic stem cells; HPCs hematopoietic progenitor cells; CB umbilical cord blood; O_2_ oxygen; UMAP uniform manifold approximation and projection; HIF hypoxia inducible factor.

Our in vivo data lead us to infer that local O_2_ affects HSC/HPC potency, particularly in cells responsible for early immune reconstitution and those that retain a more balanced myeloid/lymphoid ratio. We sought to identify subclusters of cells associated with these alterations in functional potency and determine the molecular effects of local O_2_ tensions on various subpopulations of HSCs/HPCs. We performed single cell RNA sequencing (scRNA-seq) under oxygen-controlled conditions using PIP-seq [30]. A total of four CBUs were expanded for 2 or 7 days at 5 O_2_ levels or were sequenced unmanipulated as input cells. The majority (60%) of unmanipulated CD34+ cells were transcriptomically distinct from cultured cells (Fig. 5A, Fig. S3A, Table S1) and were separated and sub-clustered for higher resolution (Fig. 5A, Fig. S3B).

Our final single cell object consisted of 26 annotated clusters of cells (Fig. 5A, Fig. S4, Table S2). Clusters comprised primarily of unmanipulated CBUs were termed “unexpanded” HSCs, MPPs, myeloid MPPs, CMPs, GMPs, and CMP-GMP transitioning. Other than unmanipulated input, only cells expanded at 1% O_2_ for 2 days maintained significant proportions of “unexpanded” transcriptomic characteristics (Fig. 5B). By day 7 of expansion, few cells in any expanded group were found in these clusters, though low O_2_ tensions still maintained higher a higher proportion of cells with these molecular characteristics (Table S2). This confirms that ex vivo expansion of CD34+ cells dramatically alters their transcriptomic profile, but this can be mitigated for short time periods by maintaining the cells at very low physiologic (1%) O_2_. We annotated cells enriched for primitive HSC/MPP markers in expanded conditions on day 2 or day 7 and termed them “expanded early HSC/MPP” (Fig. 5C). Notably, CD34+ cells expanded at lower O_2_ tensions (1–5%) exhibit a higher proportion of these cells after the full seven days of expansion compared to input and higher O_2_ levels (Table S2).

**Fig. 6:**
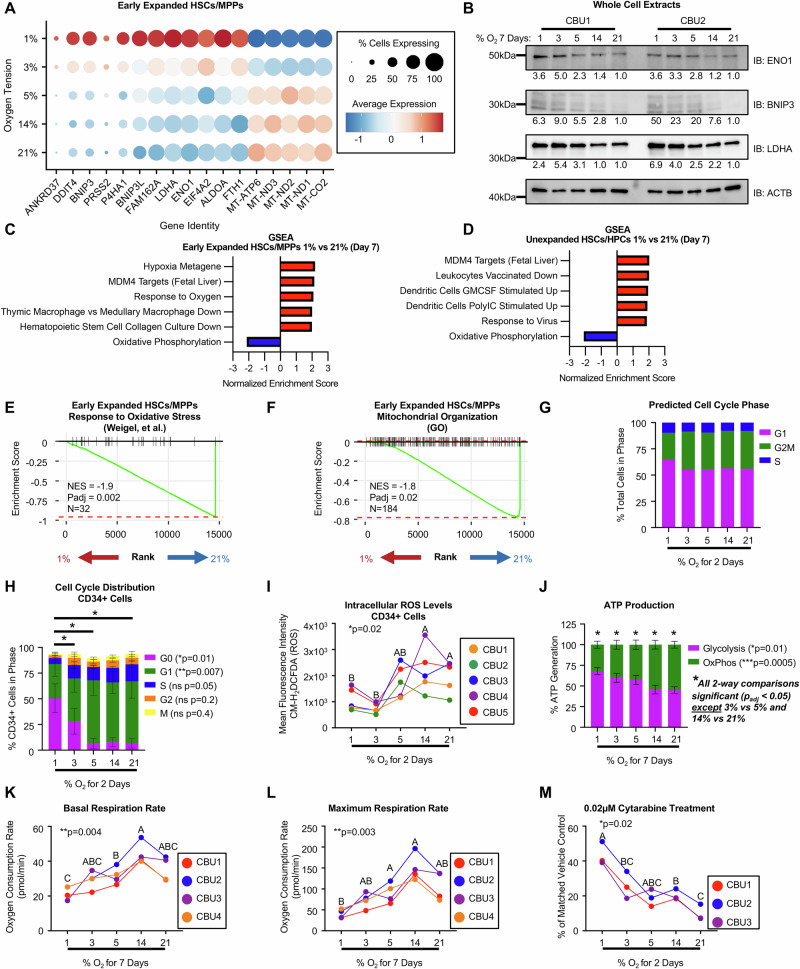
HSCs/HPCs exhibit oxidative stress associated properties in higher O_2_ tensions. **A** Representative dot plot showing genes that are up and downregulated in low and high O_2_ tensions after 7 days of culture in the Early Expanded HSCs/MPPs subcluster of cells. **B** Representative western blot of whole cell lysates using antibodies targeted against indicated proteins after 7 day expansion in the shown O_2_ conditions. Densitometry analysis of band intensity was performed and is calculated relative to the 21% bands from the same antibody blot, relative to ACTB as a normalizing housekeeping protein. The numbers from this analysis are under the images of the western blot. **C**,**D** Top ranked gene sets from GSEA analysis from the indicated cell populations cultured for 7 days in 1% vs 21% O_2_. Gene sets were first filtered by significance (p_adj_<0.05), were sorted by normalized enrichment score, and were then reduced for redundancy by removing gene sets with >50% intersection. Top five upregulated gene sets are shown; only one downregulated gene set passed the statistic filters. The full list of gene sets can be found in Table S2. **E–G** CB CD34+ cells expanded in serum free media with growth factors for 2 days (*n* = 2) or 7 days (*n* = 2) in the indicated O_2_ tensions, or unmanipulated input cells (*n* = 2) were subjected to scRNA-seq using particle-templated instant partition sequencing (PIP-seq). **E**,**F** Representative GSEA plots showing the enrichment for the indicated gene programs in 1% versus 21% O_2_ in the subcluster Early Expanded HSCs/MPPs. **G** Transcriptomic prediction of cell cycle status for all cells in the 2-day expanded groups for the indicated O_2_ tensions. **H–M** CB CD34+ cells expanded in serum free media with growth factors for 2 or 7 days in the indicated O_2_ tensions. **H** Cell cycle analysis using Ki67/DAPI flow cytometry analysis after 2 days expansion (*n* = 3). I Intracellular reactive O_2_ species (ROS) measurement after 2 days expansion using CM-H2DCFDA ROS indicator and flow cytometry analysis (*n* = 5). **J–L** Seahorse metabolic flux analysis after 7 days expansion using J) ATP rate assay to show ATP production (*n* = 4) or **K**,**L** mitochondrial stress test (*n* = 4) to show calculated basal and maximum respiration rates. **M** CD34+ cells were treated with 0.02 µM cytarabine (*n* = 3) for 2 days in variable O_2_ tensions and counted by Trypan Blue viability staining. Stats: one-way or two-way ANOVA (indicated by p-value) with post hoc comparisons indicated by compact letter display (in compact letter display, groups that are not statistically different are indicated by matching letters). Colors used are described in figure legends. HSCs hematopoietic stem cells; HPCs hematopoietic progenitor cells; CB umbilical cord blood; O_2_ oxygen; IB immunoblot; GSEA gene set enrichment analysis; ROS reactive oxygen species.

We examined genes that are altered in response to differences in local O_2_ tension, using 1% O_2_ and 21% O_2_ as differential conditions. We focused on two cell populations of interest comprised of primitive HSCs/HPCs that contain the functionally potent cells we seek to assess and that include sufficient cell numbers from each condition for differential analysis. The first was a pooled population defined by characteristics of unmanipulated CD34+ cells containing “Unexpanded” HSCs, MPPs, myeloid MPPs, CMPs, GMPs, and CMP-GMP along with MEPs, MKPs, MLPs, and ERPs (which do not clustered separately based on cell culture conditions). We also analyzed the individual cluster “Expanded Early HSCs/MPPs”. In the “Expanded Early HSCs/MPPs” cluster and the “Unexpanded” pooled population, 5 and 51 genes, respectively, were significantly altered by oxygen tension after 2 days in culture, while 16 and 79 genes were altered after 7 days of culture (Fig. 5D-E representative genes, Table S2 full list). Given the importance of Hypoxia inducible factor (HIF) proteins in regulating hypoxia responses, we examined differentially expressed genes for predicted regulation by HIF1a and HIF2a by mining previously published ChIP-seq data [36]. Across the two analyzed cell populations, 29.7% of differentially expressed genes are bound by HIF1a, with nearly half of those being co-bound by HIF2a (Fig. 5D-E, Table S2). Most (>90%) of the HIF peaks localized to genes that were only altered after 7 days of culture (Fig. 5E). These findings suggest that HIF contributes to the regulation of a subset of O_2_-dependent genes in these HSCs/HPCs subpopulations and that its influence becomes more apparent during longer-term culture. Interestingly, we observed that “Early Expanded HSCs/MPPs” exhibit O_2_-dose-dependent gene expression responses up to 5%, but at 5% O_2_ and above, they show similar expression patterns (Fig. 6A). We infer that in this cell population specifically there are molecular switches in the low physiologic O_2_ space somewhere between 3 and 5% O_2_ responsible for oxygen-dependent gene expression regulation that may or may not be HIF-dependent. By western blot we confirmed changes in expression in bulk CD34+ cells at the protein level of select O_2_ dependent molecules including enolase 1 (ENO1), BCL2 interacting protein 3 (BNIP3), and lactate dehydrogenase A (LDHA) (Fig. 6B, S5A–C). All together, these analyses reveal O_2_ dependent genes associated with potent subpopulations of cells that may be important for HSC/HPC functions.

### O_2_ affects hematopoietic cell stress

We next performed unbiased gene set enrichment analyses (GSEA). To reduce redundancy inherent in these results, we reported the top gene sets with positive and negative enrichment scores that had less than 50% overlap in their leading edge genes with other top gene sets (Fig. 6C, D). As expected, the most enriched gene programs in cells grown under low O_2_ were associated with hypoxia, and this gene program was redundant with specific pathways including HIF [37] and mechanistic target of rapamycin complex (MTORc) [38] signaling (Fig. 6C, D, S5D, E, Table S2). Interestingly, gene programs associated with the MDM4 regulator of p53 (MDM4) pathway (which has recently been implicated in hematopoietic cell potency [39]), as well as specific types of immune function and/or differentiation states (e.g., thymic macrophage vs epithelial macrophage; dendritic Cell stimulation), were also enriched in cells grown at 1% O_2_. These data suggest that hematopoietic cell function and differentiation are influenced by local O_2_ tension. In contrast, cells expanded in 21% exhibited transcriptomes enriched for genes associated with oxidative phosphorylation, which overlapped with gene programs associated with oxidative stress and mitochondrial functions, such as ATP synthesis. This pattern suggests an underlying accumulation of acute metabolic stress in cells grown at higher O_2_ tensions (Fig. 6C–F, S5D, E). Finally, cells grown at lower O_2_ levels are predicted by their transcriptomes to have a higher proportion of cells in G1 (Fig. 6G), which may also indicate quiescence.

Based on these findings, we tested whether hematopoietic cells exhibit changes in stress associated properties in different local O_2_ tensions. By flow cytometry, we analyzed the cell cycle status of CD34+ cells grown in variable O_2_ tensions. Cells grown in 1% and 3% O_2_ for 2 days had increased proportions in G0 compared to higher O_2_ tensions (Fig. 6H), confirming the transcriptomic prediction. CD34+ cells grown in 5%, 14%, and 21% O_2_ also exhibited increased accumulation of intracellular reactive O_2_ species, a marker for oxidative stress (Fig. 6I). Given the oxygen-dependent effects on reactive O_2_ species and mitochondrial gene expression (Fig. 6A, E, F, I), we next sought to examine the O_2_ associated metabolic properties of HSCs/HPCs using the Seahorse metabolic flux analyzer. ATP rate assays showed that CD34+ cells generate significantly more ATP from glycolysis in lower O_2_ tensions (1%, 3%, and 5%) compared to 14% and 21%, at the expense of ATP generated from oxidative phosphorylation (Fig. 6J). Cells grown in 1% had the lowest ratio of oxidative phosphorylation to glycolysis compared to all other expansion conditions (Fig. 6J). To further examine the oxidative phosphorylation pathway, we examined the effects of inducing acute stress using the mitochondrial stress test (Fig. S6A). CD34+ cells exhibited oxygen-dose dependent effects on mitochondrial respiration, with cells grown at 1% O_2_ exhibiting significantly lower basal and maximal respiration rates as well as lower spare respiratory capacity compared to 14% and 21% (Fig. 6K-L, Figure S6A).

These data show that CD34+ cells grown at higher O_2_ tensions are more metabolically active, more proliferative, and accumulate more stress related molecular properties compared to those maintained in lower O_2_. Thus, we hypothesized that CD34+ cells in low physiologic O_2_ are more resistant to acute toxicities. To test this, we treated CD34+ cells with the broadly cytotoxic chemotherapy cytarabine in variable O_2_ levels. CD34+ cells grown in low O_2_ tensions, particularly 1% oxygen, were highly resistant to cytarabine induced cell death compared to higher O_2_ levels, as indicated by a significantly higher percentage of viable cell number after 2 days of treatment compared to vehicle (Fig. 6M) and higher expansion over time in the presence of cytarabine (Fig. S6B). These data provide insights into hematopoietic toxicities following chemotherapy and resistance of malignant hematopoietic cells to treatment.

### O_2_ dependencies are partially regulated through MTORc

**Fig. 7:**
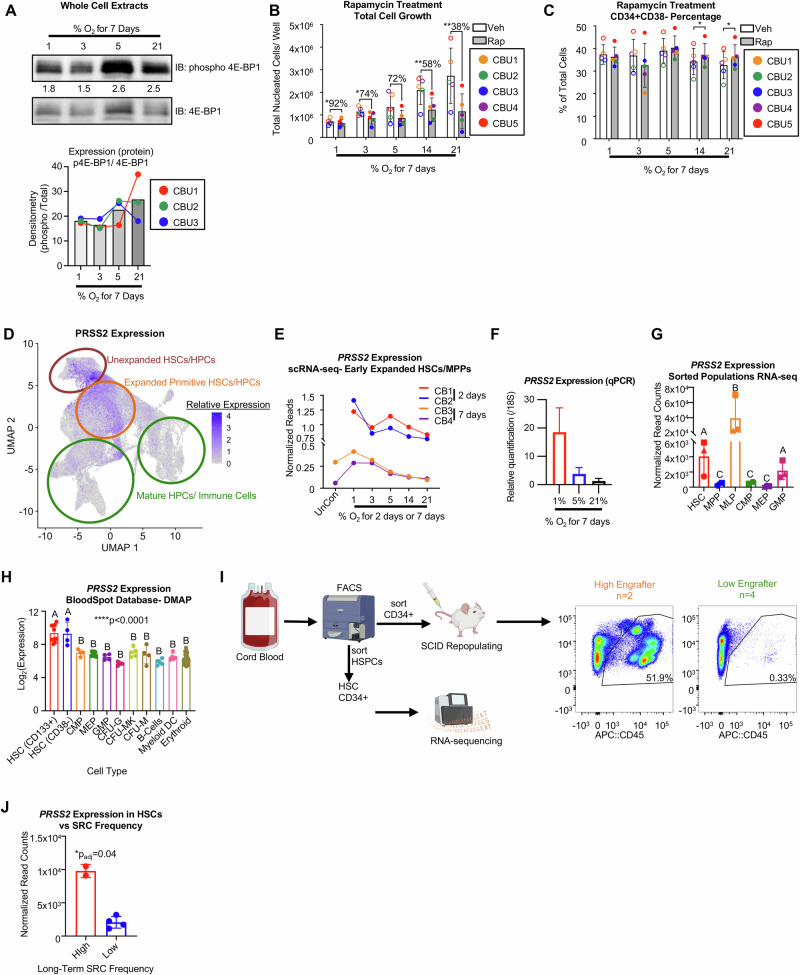
Oxygen-dependent HSC/HPC functional potency are partially regulated through MTOR and *PRSS2* predicts long-term engraftment of HSCs. **A** Representative western blot of whole cell lysates using antibodies targeted against indicated proteins after expansion in the shown O_2_ conditions. Densitometry analysis of phosphor 4E-BP1 band intensity was performed and is calculated relative to total 4E-BP1. The numbers from this analysis are under the images of the representative western blot, and all replicates (*n* = 3 cord blood units) are shown in the graph underneath the western blot. **B** Total nucleated cells and **C** CD34 + CD38- cells were enumerated by flow cytometry after 7 days of treatment with 25 nM Rapamycin in the indicated O_2_ tensions. **D** Feature plot showing *PRSS2* expression in primitive HSCs/HPCs clusters from scRNA-seq. **E** Expression of *PRSS2* in different O_2_ tensions in HSCs/MPPs by scRNA-seq. **F** qPCR measuring PRSS2 expression relative to 18S housekeeping gene of 3 technical replicates of CD34+ cells from one CBU grown at the indicated oxygen tensions. **G** Expression of *PRSS2* in immunophenotypically defined and sorted HSCs/HPCs by bulk RNA-seq. **H** Expression of *PRSS2* in HSC/HPC populations from a publicly available database. **I**,**J** *PRSS2* expression in HSCs correlated with long-term engraftment capacity in a mouse model of human CB transplantation. DESeq2 used for significance of differential expression analysis. Stats: (**B**,**C**) one-way or two-way ANOVA (indicated by p-value) with post hoc comparisons indicated by compact letter display (in compact letter display, groups that are not statistically different are indicated by matching letters). Colors used are described in figure legends. G/J DESeq2 statistical analysis. H one-way ANOVA (indicated by p-value) with post hoc comparisons indicated by compact letter display (in compact letter display, groups that are not statistically different are indicated by matching letters). HSCs hematopoietic stem cells; HPCs hematopoietic progenitor cells; CB umbilical cord blood; O_2_ oxygen; UnCon Unmanipulated control (input).

Several genes and gene programs that exhibit stabilized expression in low O_2_ tensions in HPC populations are linked to MTORc signaling, (Figs. 5D, E, 6A–D), which regulates processes critical to HSC/HPC fate including mitochondrial function, biogenesis, and degradation [40]. Paradoxically, genes altered by low O_2_ include both repressors of MTORc signaling (*BNIP3* and DNA damage inducible transcript 4 (*DDIT4*)) [41–43] and genes activated by MTORc (*LDHA* and *ENO1*) [44, 45]. We sought to determine the degree to which this pathway may contribute to the observed O_2_-dependent phenotypes. We found by western blot that canonical targets of MTOR, S6K [46] and 4E-BP1 [40] exhibited inconsistent trends toward increased phosphorylation with higher oxygenation (Fig. 7A, S7A). Despite this, CD34+ cells grown in high O_2_ in the presence of MTORc inhibitor Rapamycin consistently showed significantly decreased expansion of total nucleated cells (Fig. 7B) and modest, though significant, increases in proportions of the potent HSC/HPC enriched CD38- population (Fig. 7C). Consistent with an MTOR role in hematopoietic potency, published work showed that Rapamycin treatment increases in vivo engraftment of human CD34+ cells expanded in ambient air [47]. However, Rapamycin treatment did not affect O_2_-dependent mitochondrial respiration measured by Seahorse and did not phenocopy low O_2_ stabilization of *BNIP3*, *LDHA*, or *ENO1* at the transcription level (Fig. S7B–I). Taken together, our data suggest that MTOR plays a partial role in the observed O_2_-dependent phenotypes, and that its effects on hematopoietic potency may be mediated through non-canonical targets. The modest effects observed with MTOR inhibition further suggest that a dynamic interplay among multiple regulatory- including but not limited to pathways regulated by HIF, MDM4, and MTORc revealed by our single cell transcriptomic data- contributes to molecular O_2_ sensing and the regulation of hematopoiesis (Fig. 6C, D). Full mechanistic investigation of each O_2_ associated pathway is warranted in future studies.

### O_2_ dependent transcripts predict engraftment outcomes

We inferred based on the known phenomena of HSCs/HPCs losing overall engraftment potential during long-term ex vivo culture that “unexpanded” HSC/HPC clusters are enriched with functionally potent cells. Interestingly, in addition to known markers *THY1* and *AVP* [48, 49], unexpanded HSCs also exhibited high expression of serine protease 2 (*PRSS2*), which encodes pancreatic Trypsin 2 protein and has well described digestive functions but does not have a well characterized hematopoietic function [50, 51]. The role of *PRSS2* in HSC/HPC biology is unclear, though its hematopoietic expression has been reported. *PRSS2* expression is primarily confined to unexpanded cell clusters as well as “Expanded Early HSCs/MPPs” cluster, which we predict contains cells responsible for engraftment from expansion conditions (Fig. 7D). Further analysis of scRNA-seq data as well as qPCR data shows that *PRSS2* is stabilized in low O_2_, consistent with it being associated with increased HSC/HPC potency (Fig. 7E-F). To confirm expression of *PRSS2* in HSCs/HPCs, we sequenced immunophenotypically defined HSC/HPC subpopulations from freshly isolated CBUs and found that *PRSS2* is most highly expressed in MLPs followed by HSCs (Fig. 7G, Table S3). In independently published data, HSCs exhibit significantly higher expression of *PRSS2* compared to mature HPC populations (Fig. 7H). We hypothesized that *PRSS2* might be a transcriptional marker of HSC potency. To test this, we performed bulk RNA-seq on HSCs from biologically distinct CBUs with known engraftment outcomes in mouse models of transplantation (Fig. 7I; Table S4,S5), modeling the CD34+ and HSC transcriptomes as a function of SCID repopulating cell (SRC) frequencies. These data revealed that high *PRSS2* expression in HSCs accurately predicts high SRC frequency (Fig. 7J). Notably, the MTORc regulator *DDIT4* also trended toward higher expression in CBUs with higher SRC frequencies, but only in the broader CD34+ population (Figure S8). Whether these genes regulate hematopoiesis in an O_2_-dependent manner remains to be determined. Together, these data showed that specific O_2_ dependent genes mark potent HSCs/HPCs in context of transplantation.

## Discussion

HSCs/HPCs are exposed to a wide range of O_2_ tensions during their physiologic lifespan and when being studied or utilized ex vivo. Here we showed that HSCs/HPCs are acutely sensitive to physiologic O_2_ with regards to immunophenotype, self-renewal, and differentiation properties as well as in vivo potency and molecular profiles, particularly stress responses. All together, our data suggest that physiologic O_2_ is a critical extrinsic factor that affects the growth and function of HSCs/HPCs through regulation of stress associated molecular pathways. Multipotent hematopoietic cells remain the most quiescent, show the lowest biochemical signs of stress accumulation, and express genes associated with stem cell properties in low physiologic O_2_ tensions (1-3%), though their proliferative capacity is very specific to the specific cell subpopulation with HSC frequency highest in 5% and MPP frequency highest in 1–3%. When exposed to higher physiologic and extra physiologic tensions, HSCs expand poorly, suggesting that they are differentiating. Mature HPCs expand rapidly in higher O_2_ tensions and show properties associated with higher metabolism, rapid proliferation, and stress accumulation. Thus, HSCs/HPCs may utilize local BM O_2_ tensions to maintain the pool of HSCs or rapidly initiate differentiation and expansion of HPCs in response to different homeostatic or pathogenic states. The high O_2_ tensions these cells experience outside the BM likely intensifies HPC tendencies to proliferate, which would drive expansion of lineage defined progenitors in response to infection or wounding. Future studies should examine the O_2_ dependency of other cells of hematopoietic origin like mature lymphocytes.

Our findings have immediate translational implications. First, malignant hematopoietic cells are also exposed to variable O_2_ levels and have been shown to be affected by hypoxia [52–54]. We infer from our data that O_2_-dependent gene programs are partially responsible for chemotherapeutic resistance and can use our transcriptomic profile to identify targets for treatment sensitization. Additionally, hematopoietic cell therapies including CB transplantation are a critical tool for treating patients with hematologic disorders and some non-hematologic disorders [4]. A limiting factor in the efficacy of transplantation is the number of functionally potent HSCs/HPCs that can be delivered to the patient. Here we have identified O_2_ dependent genes that mark potent HSCs/HPCs, including *PRSS2* and *DDIT4*, which could aid in the selection of the optimal donor units that are most likely to achieve favorable outcomes. Further, oxygen-dependent gene programs, such as HIF, MDM4, MTORc signaling, and cellular stress pathways represent potential therapeutic targets to improve hematopoietic potency and HSC/HPC responses to stress. These pathways are likely dynamically interconnected and should be targeted both individually and in combinations to evaluate therapeutic efficacy. All identified O_2_-dependent genes are potentially hematopoietic regulatory factors that are understudied due to the limitations of non-physiologic culture conditions. These warrant further investigation with full mechanistic studies. Finally, understanding which conditions are best for use in ex vivo proliferation allows us to improve existing clinical protocols for hematopoietic cell therapies that require ex vivo manipulation, including CB expansion, gene editing, and the derivation of off-the-shelf immune effector therapies from HSCs [24, 55–58]. Previous studies have shown that human and mouse HSCs are best cultured in low O_2_ levels compared to higher O_2_ tensions, but there are discrepancies about how low that tension should be [26–29]. Our study provides insights into the nuanced biological O_2_ dependencies by examining source specific and cell type specific effects of different oxygenation. For example, our data suggests that very low physiologic O_2_ (1%) is ideal for maintenance of functionally potent CB HSCs and early HPCs, while mid-range O_2_ (3-5%) may be best for ex vivo expansion of early repopulating cells. Understanding the best conditions in which to expand HSCs/HPCs, as well as the best conditions to improve HPC commitment to target lineages will be critical for the improvement of these therapies. We demonstrated that BM and mPB are also sensitive to different O_2_ levels, though to different extents in different populations. Future work should optimize the leveraging of O_2_ dependencies in these donor sources for improved therapeutics.

In all, this study provides insight into how physiologic levels of O_2_ may drive changes in hematopoietic cell function during homeostasis and stress and how this can be leveraged to improve HSC/HPC potential for therapies.

## Supplementary information

Supplemental Materials, Methods, and Figures

Table S1

Table S2

Table S3

Table S4

Table S5

## Data Availability

The analyzed sequencing datasets generated during the current study are available in Mendeley, DOI: 10.17632/8zzm2nr4w6.1. Raw data and processed count files are available in the Gene Expression Omnibus (GEO) under the accession numbers GSE322748 (scRNA-seq) and GSE322893 (bulk RNA-seq). All other data are included in this article and supplementary information files or are available upon request to the corresponding authors.
