# Supplementary material for: Local oxygen tension dictates hematopoietic cell growth and potency
Source: Leukemia. 2026 May 15;40(7):1502–15. doi: 10.1038/s41375-026-02971-w (PMC13308678; doi:10.1038/s41375-026-02971-w)
Supplement: Supplementary file 1 — Supplemental Materials, Methods, and Figures [file 41375_2026_2971_MOESM1_ESM.pdf]

Detailed Methods:

CD34+ cells isolation or purchase:

Fresh CB >= 60mL in volume and <55hrs since birth were collected in 35mL of Anticoagulant Citrate Phosphate Dextrose Solution and provided by or purchased from the Cleveland Cord Blood Center. Low density CB was collected using density centrifugation with Ficoll Paque (Cytiva). CD34+ cells were isolated using magnetic anti-CD34 monoclonal antibody and magnetic selection columns (Miltinyi). CD34+ cell pellets cryopreserved in 90% FBS + 10% DMSO freezing solution and stored in liquid nitrogen. Frozen CD34+ cells derived from three distinct human bone marrow donors were purchased from STEMCELL. Frozen CD34+ cells derived from three distinct mobilized peripheral blood donors were purchased from the Fred Hutch Cooperative Center for Excellence in Hematology Hematopoietic Cell Procurement and Processing Core. Prior to use in assays, CD34+ cells were thawed rapidly by warming in a 37°C water bath with gentle agitation and DMSO was washed out.

Immunophenotyping by Cell Surface Staining and Fluorescence-Activated Cell Sorting:

$1x10^5$-$1x10^6$ freshly thawed CD34+ enriched cells or cells from CD34+ expansion were incubated in PBS with fluorophore conjugated antibodies from BioLegend targeting CD34, CD38, CD45RA, CD10, CD49f, CD90, and CD135. Cells were washed 2x with cold PBS and were fixed with 1% paraformaldehyde (Pierce) and analyzed using FACS. Positive and negative gates were drawn based on unstained and single stained controls. Cell subpopulations were defined as follows: HSC: CD34+CD38-CD45RA-CD49f+CD90+; MPP: CD34+CD38-CD45RA-CD49f-CD90-; MLP: CD34+CD38-CD45RA+CD10+; CMP: CD34+CD38+CD10-CD45RA-CD135+; MEP: CD34+CD38+CD10-CD45RA-CD135-; GMP: CD34+CD38+CD10-CD45RA+CD135+. For cell cycle analysis, cells were permeabilized and stained with KI67 and DAPI. Cell populations were annotated as follows: G0: CD34+ DAPI low KI67-; G1: CD34+ DAPI low KI67 int; S: CD34+ DAPI int KI67 int; G2: CD34+ DAPI high KI67 int; M: CD34+ DAPI high KI67 high. For ROS analysis, cells were incubated with the cell permeant ROS detector CM-H2DCFDA (ThermoFisher) diluted to 5μM at 37°C for 30 minutes, washed twice, and analyzed immediately.

Ex Vivo Expansion Assays using Total CD34+ Cells:

Thawed CD34+ cells were grown at a density of $5x10^4$ cells/mL in 1mL SFEM II (STEMCELL Technologies) supplemented with 100ng/mL recombinant human thrombopoietin (TPO), 100ng/mL recombinant human Stem cell factor (SCF), and 100ng/mL recombinant human FLT3

ligand (FLT3L) (R&D Technologies). Cultures were incubated at 37°C in a humidified incubator kept at 5% CO2 and the O2 level indicated by the experiment. These tensions were 1%, 3%, 5%, 14%, and 21% O2. On day 4 after plating, fresh media containing the same growth factors that had been equilibrated in the appropriate oxygen tension overnight was added to a total volume of 2mL. On day 7 after plating, cells were collected, nucleated cellularity was counted by Trypan Blue staining, and expanded cells were used for immunophenotyping, CFU assays, or in vivo transplantation in mice.

Ex Vivo Expansion Assays using Prospectively Sorted HSCs/HPCs:
A total of 12 freshly thawed CD34+ cells were split into n=6 pools of CB units in order to increase the likelihood of sorting rare cell populations. These 6 pools were stained for immunophenotyping as described above. Instead of being subjected to simple flow cytometry analysis, cells were subjected to cell sorting based on their indicated immunophenotype (described above). Following sorting, cells were split three ways to be grown at O_2 tensions of 1%, 5%, and 21% (there were insufficient cells for the five tensions used in other experiments). Based on cell numbers, cells were seeded at variable densities in an appropriate multi-well plate (24-well, 48-well, or 96-well) in an appropriate volume of SFEM II (STEMCELL Technologies) supplemented with 100ng/mL recombinant human thrombopoietin (TPO), 100ng/mL recombinant human Stem cell factor (SCF), and 100ng/mL recombinant human FLT3 ligand (FLT3L) (R&D Technologies). Cultures were incubated at 37°C in a humidified incubator kept at 5% CO2 and the O2 level indicated by the experiment. On day 4 after plating, fresh media containing the same growth factors that had been equilibrated in the appropriate oxygen tension overnight was added to a total volume of 2x the initial volume. On day 7 after plating, cells were collected, viable nucleated cellularity was counted by Trypan Blue staining to determine total cell number expansion (n=6). Expanded cells were used for post expansion immunophenotyping. Due to low cell numbers in rare populations of HSCs/HPCs, post expansion immunophenotyping required another round of pooling so that two expanded units were pooled for one replicate. A total of n=3 replicates (representing twelve cord blood units from the starting material) were used for immunophenotyping. Retrospectively, one independent replicate for immunophenotyping and two independent replicates from total counting were excluded from the HSC group due to detection of a contaminating cell population. This contamination was identified by outlier levels of total expansion (over 200-fold expansion specifically in 5% compared to 10-60 fold expansion in all other replicates) combined with flow cytometry

confirming 0% CD34+ cells in the expanded cell population, suggesting a CD34- cell was missorted into the wells and outcompeted all other cell types.

Ex Vivo Colony Forming Unit (CFU) Assays:

CFU-GM, BFU-E, and CFU-GEMM colony numbers were derived by plating 250 freshly thawed CD34+ cells or 250 cells from CD34+ expansion assays in 1% methylcellulose/Iscove's Modified Dulbeccos Medium (IMDM) with 30% FBS (Corning), 1 U/mL recombinant human (rh) Epogen (EPO) (Amgen), 10ng/mL rh Interleukin-3 (IL-3) (R&D Systems), 50ng rh Stem cell factor (SCF) (R&D Systems), 10ng rh Granulocyte/macrophage colony stimulating factor (GM-CSF) (R&D Systems), 2mM L-glutamine, and 0.02mM 2-Mercaptoethanol. Cultures were incubated at 37°C in a humidified environment containing 5% CO2 and the O2 level indicated by the experiment. For direct analysis of oxygen tension effects on CFU, this was 1%, 3%, 5%, 14%, and 21% O2. For post expansion CFU numbers, this was 5% O2. Colonies were scored by manual counting 12 days after plating using an inverted microscope with phase contrast (Nikon) with a 4x objective.

In Vivo Model of Oxygen Dependent HSC/HPC Potency:

7 days prior to transplantation, CD34+ cells from 3 distinct CBUs were expanded as described above in 1%, 3%, 5%, 14%, or 21% O2. 24 hours prior to transplantation, NSG mice were given a sublethal (350gy) whole body dose of gamma radiation. Irradiated mice were transplanted by tail vein injection with 2.5x104 freshly thawed unmanipulated CD34+ enriched cells or 2.5x104 CD34+ cells that had been plated in expansion plus all expanded progeny (200uL per injection). Each group (CBU + oxygen tension) contained 3 mice, for a total of n=9 mice and n=3 CBUs per expansion condition. 2 weeks, 6 weeks, 10 weeks, and 14 weeks following transplantation, <= 50uL of peripheral blood was collected from each mouse in EDTA tubes or tubes containing heparin by submandibular bleeding. 16 weeks following transplantation, mice were euthanized and bone marrow was harvested by flushing. Equivalent cell numbers from each primary recipient mouse bone marrow were pooled per group (CBU + oxygen tension) and 1x106 pooled bone marrow cells were transplanted to secondary recipient mice that had received a 350gy dose of radiation 24 hours prior. A total of n=5 mice and n=3 CBUs were used for secondary transplantations. Secondary mice were euthanized 16 weeks after transplantation and bone marrow was harvested by flushing.

Analysis of Peripheral Blood and Bone Marrow from In Vivo Studies:

1x106 flushed BM or red blood cell depleted PB cells were stained with fluorophore conjugated antibodies: at week 2 anti-CD45, anti-CD66b, and anti CD15 for neutrophil detection; at all other time points anti-CD45, anti-CD33, anti-CD19, and anti-CD3 for human chimerism and myeloid/lymphoid ratio analysis. Cells were washed 3 times, fixed, and analyzed by FACS.

Dissolved Oxygen Measurements of CB:
Dissolved oxygen was measured from plasma of pelleted cord blood using an Orion Star A323 Dissolve Oxygen Portable Meter. A total of n=70 distinct CBUs were measured.

Single Cell RNA-sequencing Library Preparation by PIP-seq:
CD34+ enriched cells were harvested following thawing, or were expanded as described above and were collected after 2 days or 7 days of expansion (2 CBUs were expanded for 2 days, 2 CBUs were harvested as input and were expanded for 7 days). Cells harvested after 2 days had media refreshed with appropriate O_2 equilibrated media one day following plating and those expanded for 7 days had media refreshed every 2 days, to maintain fresh growth factors. On the day of harvest, cells expanded in low oxygen (1%, 3%, and 5%) were harvested in a hypoxia chamber equilibrated to 3% oxygen, while unmanipulated cells and those expanded at high oxygen tensions (14% and 21%) were harvested in an ambient air biosafety cabinet. Cells were counted and equivalent numbers of cells were incubated with cell hashing antibodies (BioLegend). A different cell hashing antibody was used for each oxygen tension and one additional was used for the unmanipulated samples. Cells were washed. Equivalent numbers of cells from each condition were pooled into one tube for each CBU. At this point cells were processed in ambient air but were kept on ice as much as possible. Total exposure to ambient air prior to cell lysis was ~90 minutes. Using the PIPseq T20 kit (Fluent Biosciences), $5x10^4$ cells from each CBU was subjected to single cell barcoding using particle emulsions. Cells were then lysed and single cell gene expression libraries and cell hashtag libraries were prepared according to manufacturer's recommendations. Cell hashing libraries were spiked in at a 1:10 ratio to the gene expression libraries and samples were submitted for sequencing to the Indiana University Center for Medical Genomics. 2-day expanded samples were sequenced for 2.5billion paired end reads on a NovaSeq 6000. Input and 7-day expanded samples were sequenced for 2.5billion paired end reads on a NovaSeqX Plus. Raw fastq files passing quality filters were obtained from the Center for further analysis.

scRNA-sequencing Analysis:

Raw fastq files were aligned to the human genome and assigned to cell barcodes and cell hashtags using PIPSeeker software (FluentBiosciences). Data was imported into R using Seurat. Remaining analyses were performed using Seurat v5. Data were normalized using SCTransform and apoptotic cells were controlled for using mitochondrial read percentages. After observing clear batch effects, the four different CBUs were integrated using Harmony. Cells lacking a hashtag or containing multiple hashtags were removed from analysis. Principal components were determined computationally. Clustering resolution was determined by comparing several different resolutions and selecting 1.0, which best separated several clusters of interest. Annotation was performed by finding markers unique to each cluster compared to all other clusters pooled, as well as by direct comparison of each cluster to every other cluster, and then by manual annotation. Full details on genes used to make annotation decisions are found in the publicly available R Script on Mendeley. Markers that define each annotated cluster are found in Supplemental Table 6.

Seahorse Metabolic Analysis:

CD34+ enriched cells were expanded as described above. Cells were washed twice with de-OCR media (Agilent Seahorse XFe RPMI media supplemented with 10mM Glucose, 1mM Pyruvate, and 2mM Glutamine). 5x104 cells were adhered to the plate using CellTak (Corning). Plates were subjected to either the ATP Rate Assay or the Mitochondrial Stress Test Assay on a Seahorse XFe96 metabolic flux analyzer, according to manufacturer recommendations. FCCP was used at 1µM.

Chemosensitivity Assays with Cytarabine:

CD34+ enriched cells from CB were expanded as described above, in the presence or absence (DMSO vehicle) of 0.02µM Cytarabine. Cells were counted with Trypan Blue,

Western Blot Protein Expression Analysis:

$1x10^5$ cells were lysed for 30 minutes on ice using RIPA buffer (ThermoFisher) containing Roche EDTA-free protease inhibitor tablets and cleared by high speed centrifugation. Lysates were run on a 4-20% denaturing Mini-Protean TGX Gel (BioRad). Proteins were transferred to 0.45µm nitrocellulose membranes. Membranes were blocked in Blocking Buffer or BSA + 0.05% Tween-20, and blotted with antibodies from Cell Signaling Technology anti-ENO1 (cat # 3810S), anti-BNIP3 (cat # 44060S), anti-LDHA (cat # 2012S), anti-p4EBP1 (cat # 2855T), anti-4EBP1 (cat # 9452S), anti-ACTB (cat # 4967S) or from Abcam anti-S6K (cat # AB184551), anti-pS6K

(cat # AB109393) followed by anti-rabbit-HRP secondary. Bands were detected with Femtoluminescent or Picoluminescent substrate (Pierce) and imaged on a ChemiDocX (BioRad). Densitometry analysis was performed using ImageLab software for relative quantification using ACTB as a normalizing protein or total target protein as a normalization factor for target phosphorylated proteins.

Rapamycin Treatment of CB:
CD34+ enriched cells from CB were expanded as described above, in the presence or absence of 25nM Rapamycin (DMSO vehicle control). Cells were then analyzed for immunophenotype or were used in Seahorse assays as described above.

Immunophenotypic HSCs/HPCs transcriptomics:
CD34+ enriched cells were thawed as described above and stained for immunophenotyping as described above. HSCs, MPPs, MLPs, CMPs, MEPs, and GMPs were sorted by FACS from three distinct CBUs and collected directly in RLT Lysis Buffer. After collection of >= $5x10^2$ cells, tubes were vortexed for 30 seconds, snap frozen, and stored in -80°C.

Long Term Engraftment/ SCID Repopulating Cell Frequency transcriptomic model:
Immunophenotypically defined HSCs or CD34+ cells were stained with fluorescently conjugated antibodies as described above and were sorted by FACS from three distinct CBUs and collected directly in RLT Lysis Buffer. After collection of >= $5x10^2$ cells, tubes were vortexed for 30 seconds, snap frozen, and stored in -80°C. The remainder of CD34+ enriched cells from the same CBUs were used for limiting dilution SCID repopulating assay. 24 hours prior to transplantation, NSG mice were given a sublethal (350rads) whole body dose of radiation. Irradiated mice were transplanted by tail vein injection with varying cell doses of $1x10^3$, $5x10^3$, or $10x10^3$ CD34+ enriched cells, 200uL per injection. Each group (cord blood unit + cell dose) contained 7-8 mice.

20 weeks following transplantation, mice were euthanized and BM cells were harvested by flushing the right femur. BM cells were counted. $3x10^6$ cells were then stained with APC anti-humanCD34 to measure human chimerism. For SCID repopulating cell (SRC) frequency calculations, mice with > 0.2% human chimerism in the bone marrow of recipient mice at week 20 after transplantation were considered responders. This value is 2.15-fold higher than background observed in an untransplanted control. SRC frequencies were determined using

Extreme Limiting Dilution Analysis (ELDA) software available from Walter+Eliza Hall Bioinformatics (https://bioinf.wehi.edu.au/software/elda/) (1). SRC frequencies were back-calculated based on CD34+ purity of the CD34+ enriched cells used for transplantation to account for differences.

RNA Extraction and Library Prep for Bulk Transcriptomics models:

For collection of RNA, frozen tubes were gently thawed at 4°C. Tubes were then vortexed again for 30 seconds and allowed to sit at room temperature for 5 min to ensure complete lysis. RNA was then harvested per manufacturer instructions using the Qiagen RNeasy Micro Plus Kit. High quality RNA (RIN => 8) was used to prepare amplified libraries either with the Indiana University Center for Medical Genomics using the SMART-Seq v4 Ultra Low Input RNA Kit (Takara) or in lab with the NEBNext Ultra Low Input RNA Sequencing Kit (NEB). mRNA libraries were submitted to the Indiana University Center for Medical Genomics and were sequenced on a NovaSeq v1.5 S4 (200 cycles kit) for paired end sequencing. Raw fastq files passing quality filters were obtained from the Center for further analysis.

RNA Extraction and qPCR for Gene Expression Analysis:

CD34+ cells from n=3 distinct cord blood units were plated in expansion conditions as described above with or without indicated treatments. Cells were harvested, washed, and lysed in RLT Lysis Buffer. Tubes were vortexed for 30 seconds, snap frozen, and stored in -80°C. For collection of RNA, frozen tubes were gently thawed at 4°C. Tubes were then vortexed again for 30 seconds, passed through a >= 20g needle 5 times, and allowed to sit at room temperature for 5 min to ensure complete lysis. RNA was then harvested per manufacturer instructions using the Qiagen RNeasy Micro Plus Kit. cDNA was then prepared using SuperScript IV kit from Invitrogen. qPCR was performed using SYBR Green 2X master mix from ThermoFisher on a QuantStudio 6 Flex Instrument. Housekeeping gene used for normalization was 18S. Primer sets used were as follows. 18S Forward- 5’ TAGGAATTGTAGTTCATGAGCGTG 3’; 18S Reverse- 5’ GGTCAGACGGGTAATGTGC 3’; BNIP3 Forward- 5’ AGCAATAATGGGAACGGGGG 3’; BNIP3 Reverse- 5’ TCTTGGAGCTACTCCGTCCA 3’; LDHA Forward- 5’ GGATCTCCAACATGGCAGCCTT 3’; LDHA Reverse- 5’ AGACGGCTTTCTCCCTCTTGCT 3’; ENO1 Forward- 5’ AAAGCTGGTGCCGTTGAGAA 3’; ENO1 Reverse- 5’ GGTTGTGGTAAACCTCTGCTC 3’

Bulk RNA-sequencing Analysis

Fastq files were analyzed for quality using FastQC. Reads were trimmed of adapters and low-quality reads using Cutadapt with the arguments -a CTGTCTCTTATA -A CTGTCTCTTATA --nextseq-trim=20 --minimum-length 50. Raw genome files downloaded from GENCODE (GRch38 version 41) were used as input to generate genome indices with STAR alignment software using the arguments –sjdbOverhang 99. Trimmed reads were aligned to the genome using STAR with the arguments --readFilesCommand zcat --outSAMtype BAM SortedByCoordinate --outSAMunmapped Within. Aligned reads were assigned to exons and annotated by gene using HTSeq with the arguments -s no -r pos -f bam. Alternatively to STAR and HTSeq, genes were aligned to the human genome and assigned to genes using Kallisto. Gene counts were normalized and differential expression was analyzed using DESeq2 R package. Sex of the baby that the CBU came from was inferred by expression of *XIST* (female specific transcription) and *DDX3Y* (male specific transcription) so that sex can be controlled for in the model where appropriate. The design used to examine differential expression between immunophenotypically defined HSCs/HPCs was ~CBU + celltype. The design used to examine differential expression between early engrafted/ homed cells and input was ~CBU + sampleType. The design used to examine differential expression between High engraftment/SRCs and Low engraftment/SRCs was ~Sex + Response. Gene set enrichment analysis was performed using test statistics generated by DESeq2 against curated datasets from MSigDB c2, c5, and c6 (downloaded from Walter+Eliza Hall Bioinformatics resource https://bioinf.wehi.edu.au/MSigDB/v6.1/) with fgsea R package with the arguments minSize = 50, maxSize = 500 and adjusted p-value cutoff of 0.05.

Statistical Analyses for Non-Sequencing Approaches

All appropriate statistical analyses were determined prior to performing experiments. For nearly all *ex vivo* studies one-way ANOVA was performed with post hoc Tukey or Sidak multiple comparisons. For Rapamycin treatment studies, two-way Anova was performed with post hoc Tukey's multiple comparisons for Vehicle vs Treated. For in vivo studies, mixed-effects linear modeling was performed to assess differences in human chimerism corrected for CBU, using CBU as a randomly sampled variable and oxygen tension as the fixed variable. Pairwise comparisons used Tukey corrections.

Description of Supplemental Tables:

Supplemental Table 1. scRNA-seq summary. Separate excel file that contains the most pertinent data from the scRNA-seq data that was discussed in this study. scRNA-seq raw data and processed Seurat object will also be made publicly available.

Supplemental Table 2. Distribution of cells in each annotated cluster as a fraction of total sequenced cells in each sample. Separate excel file. Sheet 1 shows the frequencies of cells found in the indicated annotated cell cluster from each sample (i.e., Input, 1% O_2 2 days, 1% O_2 7 days, 3% O_2 2 days, etc.) calculated as a proportion of the total cells in that sample. Red-Blue coloring is indicative of column z-scores, where deep red indicates a high proportion of cells found in that cluster and dark blue indicates a low proportion of cells found in that cluster. Sheet 2 shows all differentially expressed genes from the analysis in Figure 5 of 1% vs 21% for two different cell populations at Day 2 or Day 7 of culture. HIF bound genes are also annotated. Sheet 3 shows all GSEA results and sheet 4 shows reduced redundancy GSEA up to the top ten up or downregulated gene sets.

Supplemental Table 3. RNA-seq summary for sorted HSPCs. Separate excel file that contains a sheet for normalized read counts for all samples and for all differential expression analyses discussed in this study.

Supplemental Table 4. SRC calculations for transcriptomic analysis of engraftable HSCs. Separate excel file showing the values input into the ELDA model (https://bioinf.wehi.edu.au/software/elda/index.html)(1) and the calculated lower and upper limits for SCID repopulating cell (SRC) frequencies as well as the estimated SRC frequency for each CBU. Mean value of estimated SRC frequencies for all samples was 8.E-4. High SRC frequency was assumed for all CBUs that had greater SRC frequency than the mean.

Supplemental Table 5. RNA-seq summary for SRC model. Separate excel file that contains a sheet for normalized read counts for all samples and for differential expression analyses results.

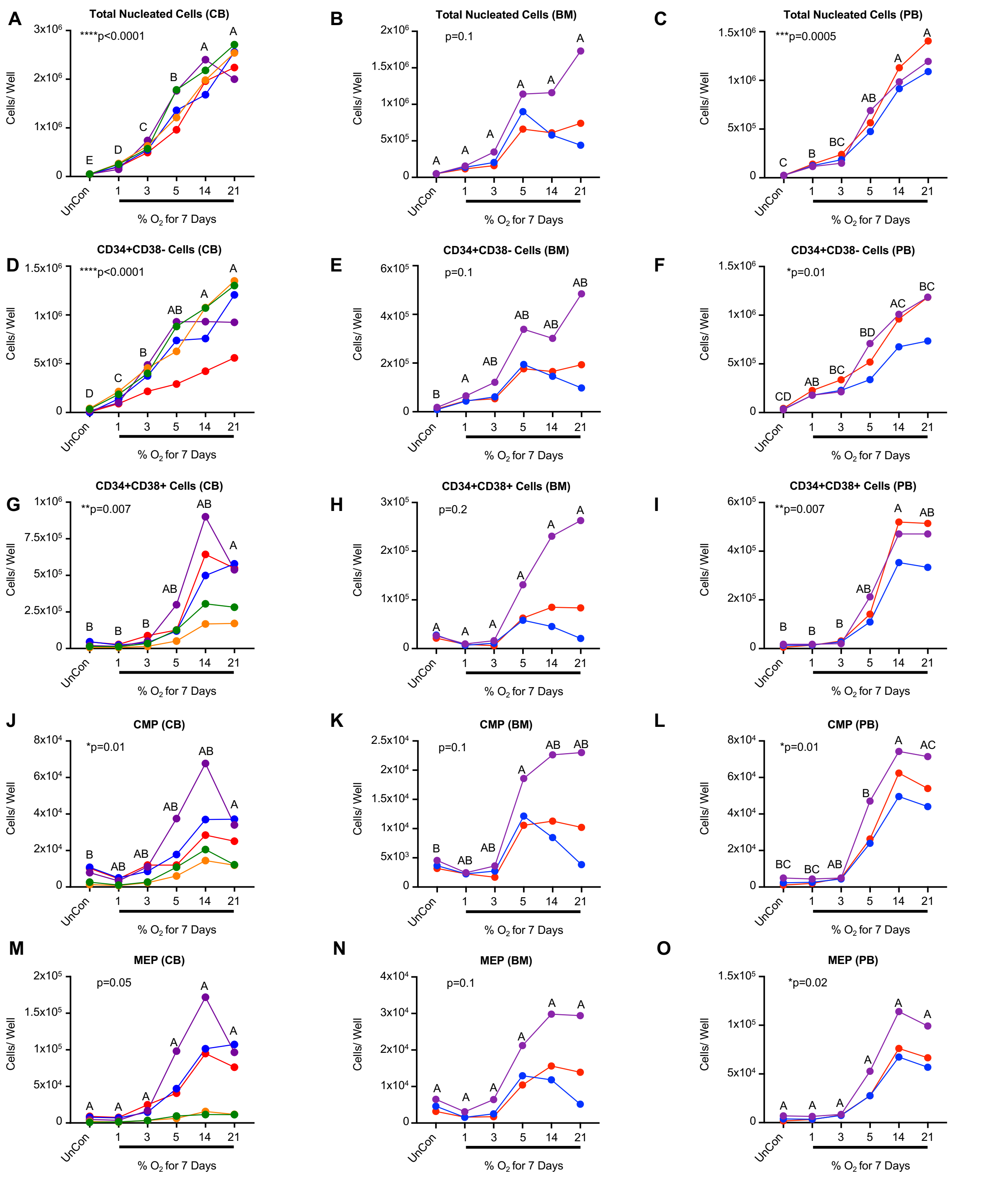

Supplemental Figure 1 (Related to Figure 2-3). A-O) Cord blood (CB), bone marrow (BM), or mobilized peripheral blood (PB) CD34+ cells were expanded in serum free media with growth factors for 7 days in the indicated oxygen tensions. Cells were then analyzed for enumeration of the indicated immunophenotypically defined population by flow cytometry (CB n=5, BM/PB n=3). Stats: one-way ANOVA (indicated by p-value) with post hoc comparisons indicated by compact

letter display (in compact letter display, groups that are not statistically different are indicated by matching letters). Each point/color indicates distinct biological donor sources as in Figures 2/3. UnCon = Unmanipulated control.

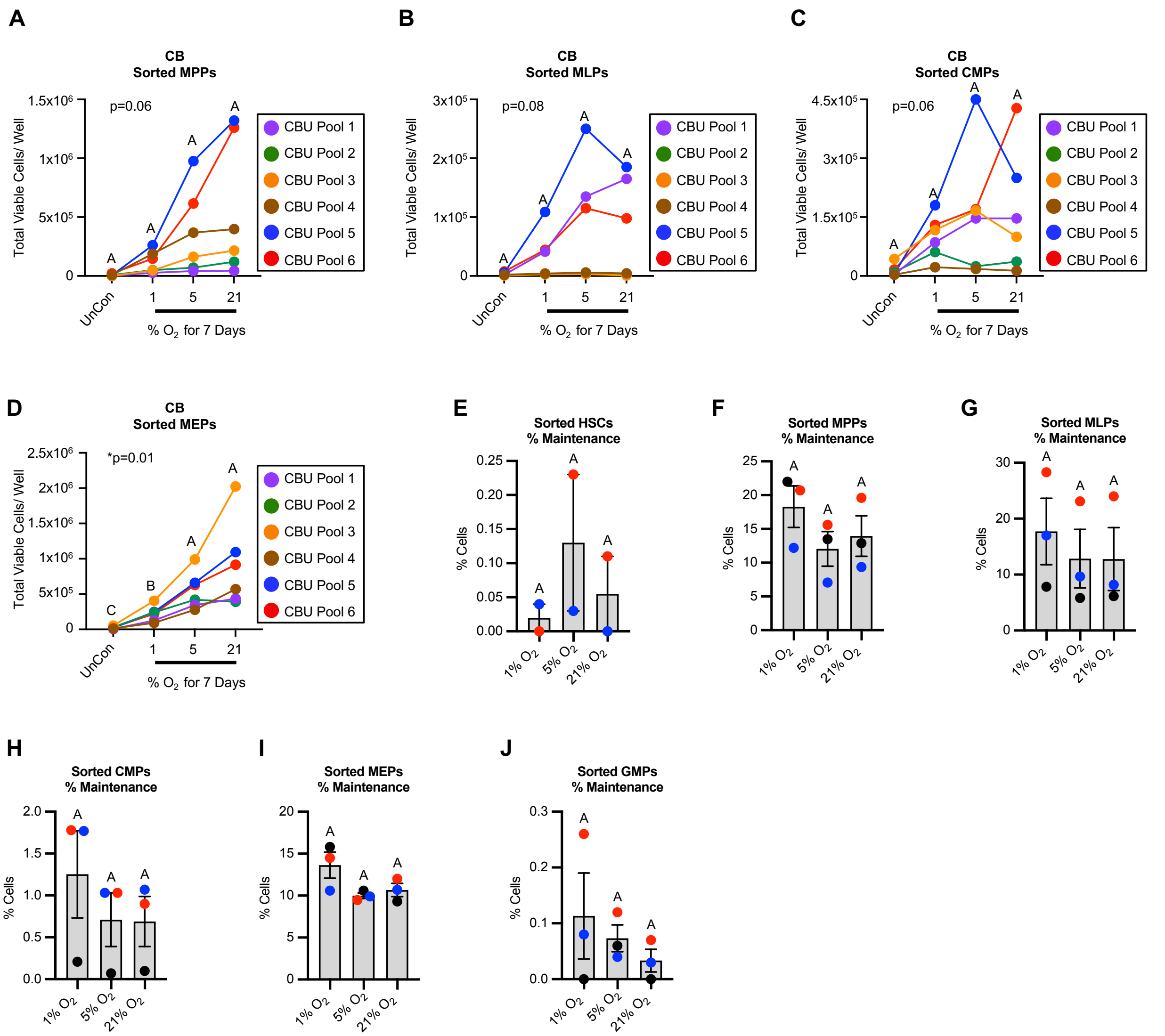

Supplemental Figure 2 (Related to Figure 2-3). A-J) Cord blood (CB) CD34+ cells were sorted prospectively prior to any culture into the indicated HPC populations. Due to cell number limitations in these rare HSC/HPC populations sorted populations were pooled so that each expansion was seeded as a pool of two distinct CB units. Sorted populations were then expanded in serum free media with growth factors for 7 days in the indicated oxygen tensions. A-D) Cells were then analyzed for total viable cell expansion by counting using a hemacytometer (n=6 CB pools). E-J) Percent of cells that were immunophenotypically defined as the same cell population that was initially sorted was determined by immunophenotyping and flow cytometry. Due to cell number limitations in these rare HSC/HPC populations, post expansion flow cytometry was performed by pooling two expansions each (n=3 pools of expansions). One independent replicate from the HSC analysis was excluded due to detection of a contaminating cell population. This contamination was identified by outlier levels of total expansion (over 200-fold expansion specifically in 5%) combined with flow cytometry confirming 0% CD34+ cells in the expanded cell population. Stats: one-way ANOVA (indicated by p-value) with post hoc comparisons indicated by compact letter display (in compact letter display, groups that are not statistically different are indicated by matching letters). Each point/color indicates distinct biological donor sources as in Figures 2/3. UnCon = Unmanipulated control.

A

	input_7d	1perc_2d	1perc_7d	3perc_2d	3perc_7d	5perc_2d	5perc_7d	14perc_2d	14perc_7d	21perc_2d	21perc_7d
0	132	1290	375	1307	356	1213	376	1245	259	1212	239
1	29	268	237	1398	234	1273	317	1362	252	1462	244
2	178	471	508	617	561	591	576	618	538	538	523
3	50	692	322	665	367	676	333	851	348	809	363
4	29	503	259	917	270	714	325	677	366	828	389
5	1822	2784	24	221	11	163	9	271	7	187	5
6	24	1868	171	656	157	564	159	758	160	672	159
7	445	350	225	571	273	574	362	529	494	549	495
8	7	560	122	790	118	675	108	769	105	763	121
9	25	273	72	770	89	730	95	883	72	789	91
10	82	175	278	232	321	191	393	224	535	220	609
11	35	55	213	172	288	125	519	161	640	162	672
12	759	22	215	128	225	97	202	76	316	127	330
13	20	212	58	517	70	563	93	483	95	574	101
14	2431	98	0	6	0	12	0	11	0	12	0
15	41	364	84	368	86	295	85	332	69	298	59
16	28	141	105	285	89	273	136	362	110	334	127
17	10	319	81	337	83	270	109	280	114	304	118
18	160	42	334	129	253	106	191	125	179	152	221
19	29	18	363	20	358	11	283	12	291	14	364
20	358	11	247	48	211	47	121	37	119	47	136
21	10	2	161	3	201	4	208	7	351	7	426
22	58	215	36	100	43	159	56	186	56	179	62
23	273	8	121	48	124	46	99	58	98	49	103
24	15	93	42	162	57	161	57	150	66	139	72
25	26	76	50	63	67	61	100	75	91	73	88
26	19	77	20	88	24	78	28	82	35	74	34
Total	7095	10987	4723	10618	4936	9672	5340	10624	5766	10574	6151

B

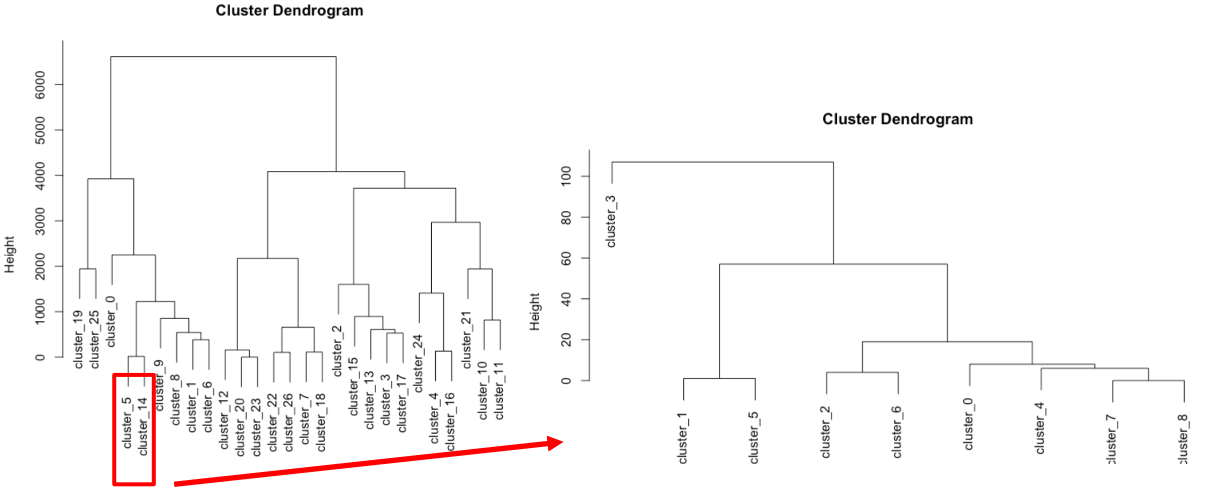

Supplemental Figure 3 (Related to Figure 5). A-B) CB CD34+ cells expanded in serum free media with growth factors for 2 days (n=2) or 7 days (n=2) in the indicated oxygen tensions, or unmanipulated input cells (n=2) were subjected to scRNA-seq using PIP-seq. A) Shown are the frequencies of cells found in the indicated initial cell cluster from each sample (i.e., Input, 1% O_2 2 days, 1% O_2 7 days, 3% O_2 2 days, etc.) calculated as a proportion of the total cells in that sample. Red-Blue coloring is indicative of column z-scores, where deep red indicates a high proportion of cells found in that cluster and dark blue indicates a low proportion of cells found in

that cluster. B) Dendrogram showing similarity scores between the different unannotated clusters. This dendrogram was used for direct comparison between clusters to perform manual annotation. Clusters 5 and 14 were subclustered due to most input cells being contained in these clusters to get a higher resolution of that sample.

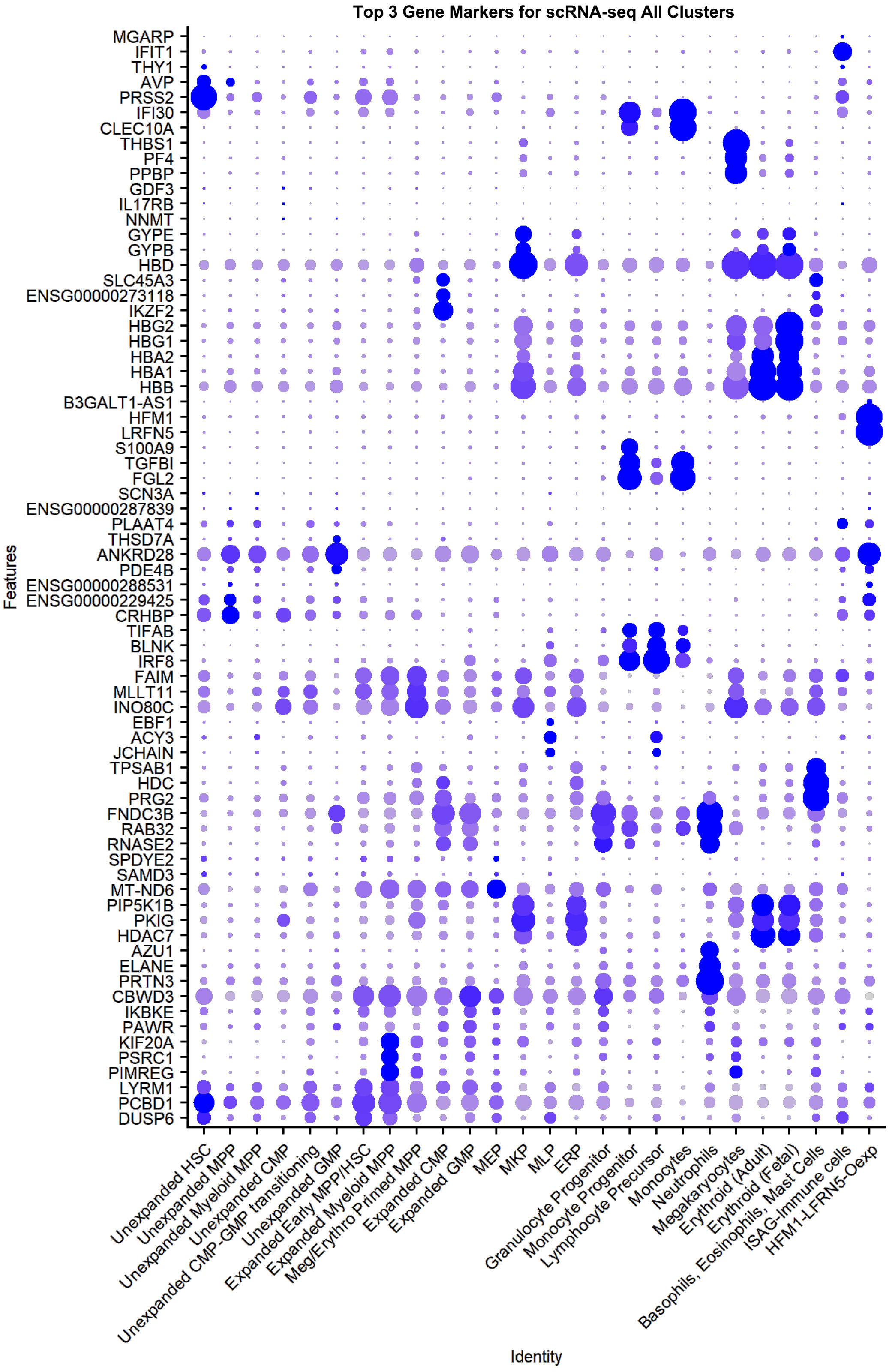

Supplemental Figure 4 (Related to Figure 5). Dot Plot showing top 3 gene markers used to annotate all cell clusters.

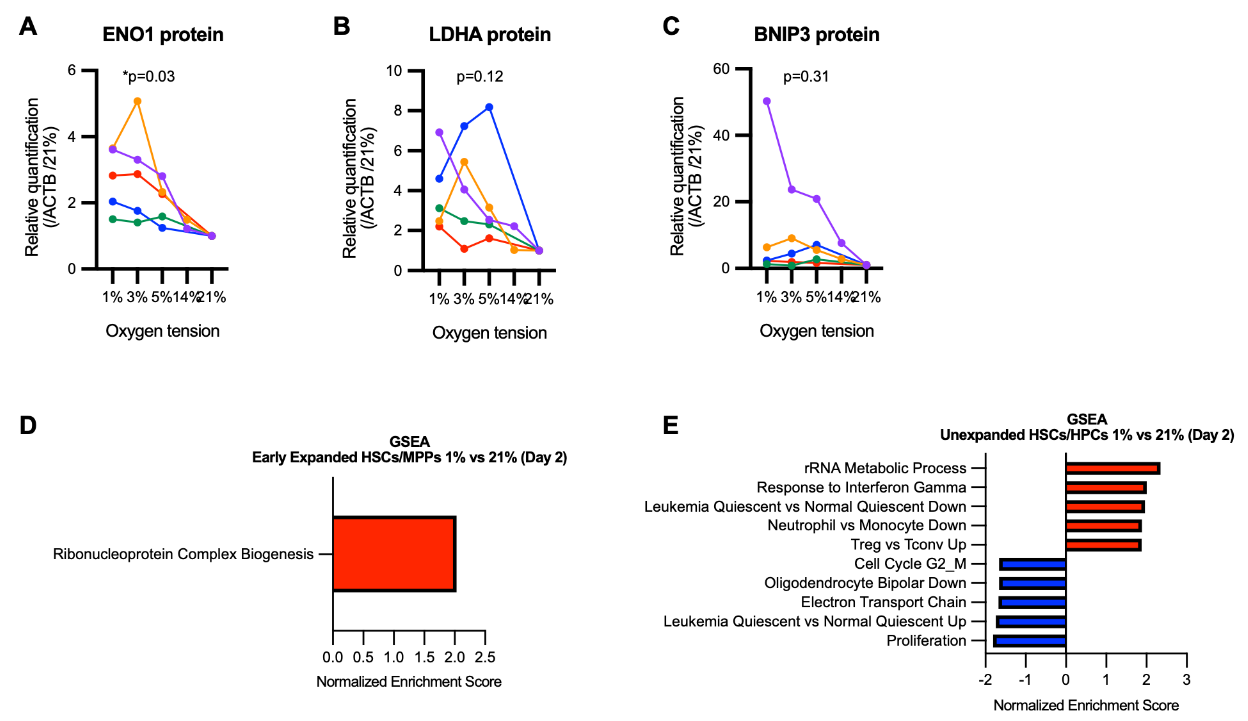

Supplemental Figure 5 (Related to Figure 6). A-C) Densitometry analysis of western blots immunoblotted with antibodies targeting the indicated protein after whole cell lysates were separated by SDS-PAGE (n=5 CB units). Values shown are normalized relative to ACTB protein levels and 21% bands. D) Stats: one-way ANOVA (indicated by p-value). There were no significant pairwise comparisons identified by post hoc analysis. D-E) Top ranked gene sets from GSEA analysis from the indicated cell populations cultured for 2 days in 1% vs 21% O_2. Gene sets were first filtered by significance (p_{adj}<0.05), were sorted by normalized enrichment score, and were then reduced for redundancy by removing gene sets with >50% intersection. Top 5 upregulated gene sets are shown; only one downregulated gene set passed the statistic filters.

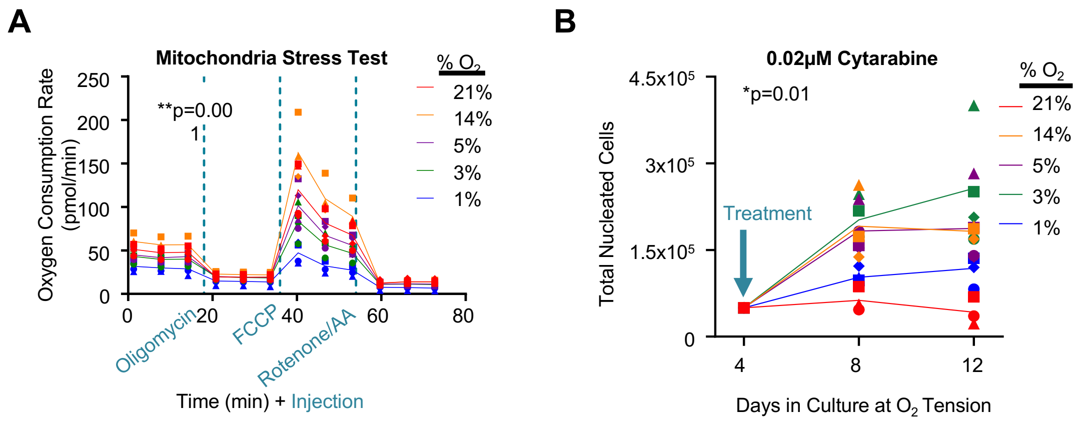

Supplemental Figure 6 (Related to Figure 6). A) Seahorse metabolic flux analysis after 7 days expansion using mitochondrial stress test (n=4) to show oxygen consumption rate following injections of Oligomycin, FCCP, and Rotenone/ Antimycin A. B) CD34+ cells were treated with 0.02μM cytarabine (n=3) for 8 days after equilibrating for 4 days in variable O_2 tensions and were counted for total nucleated cellularity. Stats: one-way ANOVA (indicated by p-value). Individual points indicate different CB donor units.

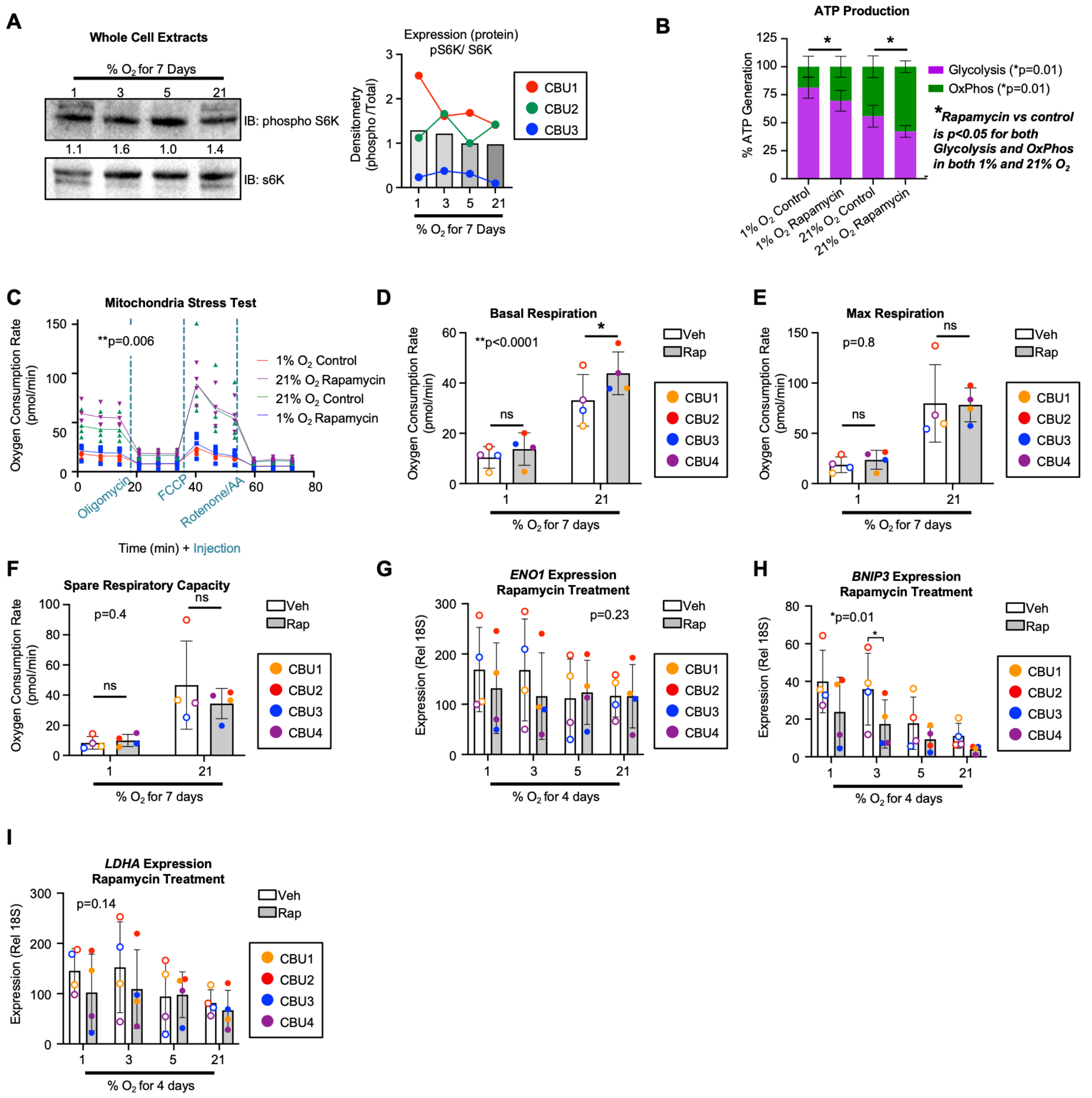

Supplemental Figure 7 (Related to Figure 7). CB CD34+ cells expanded in serum free media with growth factors for 7 days (A-F) or 4 days (G-I) in the indicated O_2 tensions in the presence or absence of 25nM Rapamycin as indicated. A) Representative western blot of whole cell lysates using antibodies targeted against indicated proteins after expansion in the shown O_2 conditions. Densitometry analysis of phospho S6K band intensity was performed and is calculated relative to total S6K. The numbers from this analysis are under the images of the representative western blot, and all replicates (n=3 cord blood units) are shown in the graph to the right of the western blot. B) ATP rate assay to show ATP production (n=4). C-F) Seahorse metabolic flux analysis after 7 days expansion using mitochondrial stress test (n=4) to show oxygen consumption rate following injections of Oligomycin, FCCP, and Rotenone/ Antimycin A. G-I) qPCR analysis of the indicated gene expression using 18S as a normalizing housekeeping gene. Stats: Two-way ANOVA (indicated by p-value) followed by post hoc pairwise comparisons. Individual points indicate different CB donor units.

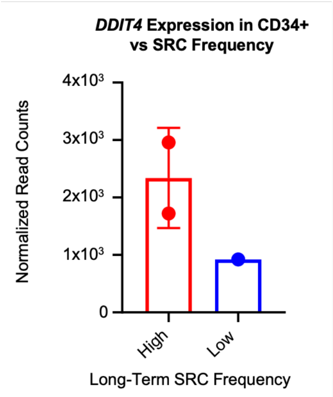

Supplemental Figure 8 (Related to Figure 7). DDIT expression in CD34+ cells correlated with long-term engraftment capacity in a mouse model of human cord blood transplantation. DESeq2 used for significance of differential expression analysis, not statistically significant due to low sample size.

Supplemental Material References

1. Y. Hu, G.K. Smyth, ELDA: extreme limiting dilution analysis for comparing depleted and enriched populations in stem cell and other assays. *J. Immunol. Methods* **347**, 70–78 (2009).
